# The Assembly of Porphyrin Systems in Well-Defined Nanostructures: An Update

**DOI:** 10.3390/molecules24234307

**Published:** 2019-11-26

**Authors:** Gabriele Magna, Donato Monti, Corrado Di Natale, Roberto Paolesse, Manuela Stefanelli

**Affiliations:** 1 Dipartimento di Scienze e Tecnologie Chimiche, Università di Roma Tor Vergata, via della Ricerca Scientifica, 1; 00133 Rome, Italy; gabriele.magna@uniroma2.it (G.M.); monti@stc.uniroma2.it (D.M.); roberto.paolesse@uniroma2.it (R.P.); 2 Dipartimento di Ingegneria Elettronica, Università di Roma Tor Vergata, via del Politecnico, 1; 00134 Roma, Italy; dinatale@ing.uniroma2.it

**Keywords:** porphyrin, self-assembly, aggregation, nanostructures, soft-matters, supramolecular polymerization, chirality

## Abstract

The interest in assembling porphyrin derivatives is widespread and is accounted by the impressive impact of these suprastructures of controlled size and shapes in many applications from nanomedicine and sensors to photocatalysis and optoelectronics. The massive use of porphyrin dyes as molecular building blocks of functional materials at different length scales relies on the interdependent pair properties, consisting of their chemical stability/synthetic versatility and their quite unique physicochemical properties. Remarkably, the driven spatial arrangement of these platforms in well-defined suprastructures can synergically amplify the already excellent properties of the individual monomers, improving conjugation and enlarging the intensity of the absorption range of visible light, or forming an internal electric field exploitable in light-harvesting and charge-and energy-transport processes. The countless potentialities offered by these systems means that self-assembly concepts and tools are constantly explored, as confirmed by the significant number of published articles related to porphyrin assemblies in the 2015–2019 period, which is the focus of this review.

## 1. Introduction

The aggregation of porphyrins and metallo-porphyrins as well as their natural or synthetic derivatives is an important issue that has been actively studied for many decades [1,2,3,4]. This class of tetrapyrrolic macrocycles is ubiquitous in nature, and represents the pivotal gear of biological systems that play an important role in many in vivo processes. For example, specific aggregates of chlorophyll molecules, firmly held together in a unique spatial arrangement by a non-covalent framework of proteins, are responsible for light harvesting and electronic energy transport to the reaction center of photosynthetic organisms, thereby leading to the high efficiency of natural photosynthesis [5]. As above-mentioned, aggregates of water-soluble porphyrins and metalloporphyrins have received great attention in recent years in light of their similarities in structure and properties to those of chlorophyll aggregates involved in photosynthetic processes [6,7]. Moreover, the possibility of self-organizing these bio-inspired dyes into various well-defined nanoarchitectures [8] is being thoroughly explored in material science due to their important applications [9,10,11]. These tetrapyrrolic macrocycles are indeed optimal building blocks for the smart assembly of supramolecular systems. Importantly, the structural features of these aromatic platforms can be shaped virtually at will by well-established synthetic methodologies, allowing for the rational design of non-covalent interactions (i.e., π–π stacking, hydrogen-bonding, metal coordination, hydrophobic effect, and electrostatic forces), and leading to porphyrin suprastructures with the desired properties and functions. Accordingly, the achievement of these soft materials aimed at specific applications such as the photodynamic therapy of tumors [12,13,14,15,16,17], organic photovoltaics, photocatalysis, and sensors is abundantly described in the literature [18,19,20,21,22]. Since the early seminal works of Robert Pasternack and others, it is clear that the propensity of porphyrins to form definite aggregates is determined by their physical properties, in terms of presence and charge type, the nature of peripheral groups and central metal atom, and its coordination state. Another important issue is the nature and the composition of the media in which the macrocycles are solubilized such as polarity, hydrogen-bonding ability, ionic strength, temperature, and pH. Quite recently, it has been demonstrated that the selection of the final structural features and properties of these supramolecular assemblies are not only dictated by the reaching of a thermodynamic equilibrium state, but the desired aggregation route can be rationally biased by suitable preparation methodologies or ancillary conditions, leading to different target structures (i.e., J- or H-aggregates, nanoparticles, nanofibers, nanosheets, and 3D morphologies) starting from the same building blocks [23,24]. Of note, by implementing elements of chirality on these porphyrin-based supramolecular systems, it is possible to further extend the potential of these assemblies in the mentioned areas, resulting, for instance, in particularly appealing asymmetric synthesis [25] and enantioselective sensing fields [26,27] Since the tremendous importance of chirality, being strictly related to the emergence and the evolution of life (biomolecular homochirality) [28,29], chiral porphyrin-based suprastructures have been deeply investigated [30,31]. 

In this report, we would like to review the latest developments related to the preparation, characterization, and application of porphyrin-based assemblies, covering in large part the last five years, with the aim to provide an update on the different aspects of the controlled spatial arrangement of porphyrins (preparation protocols, polymerization kinetics and mechanisms, micro- and nanostructure surface morphology, and so on) in elaborated architectures of theoretical and practical interest. The abundant literature has been divided in different sections based on the preparation methodology used. 

## 2. Solvent-Driven Aggregation Protocol

The aggregation of porphyrin macrocycles can be efficiently induced in mixed solvent mixtures by the so-called “good-bad” solvent protocol, consisting on solubilizing the compounds in a “good” solubilizing solvent, followed by the addition of a proper amount of a “bad” solvent, where typically the macrocycle is insoluble, even at low concentrations. Several parameters can be varied to drive the formation of ordered suprastructures with a definite morphology, as the solvent composition (i.e., type and ratio between the good and bad solvents, concentration, and order of addition of the solutions). In this section, recent examples exploiting this methodology to achieve different porphyrin-based assemblies at different length scales are reported. 

**Figure 1:**
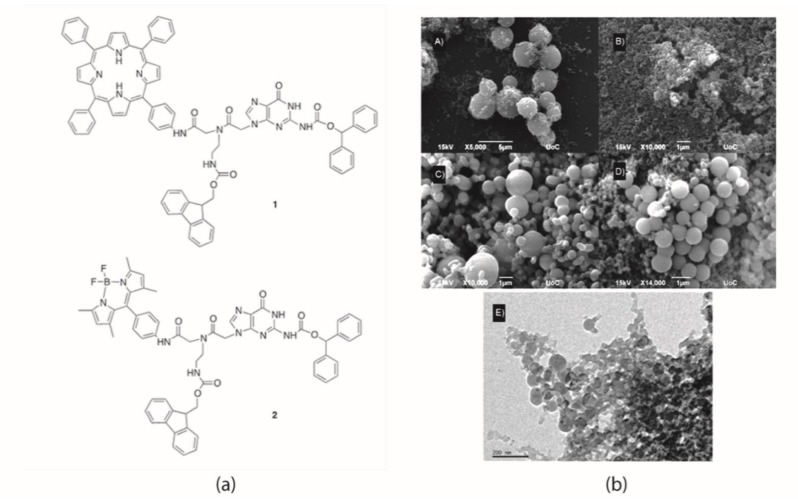
(**a**) Molecular structure of hybrids **1** and **2** studied by Coutsolelos and coworkers [15]. (**b**) Self-assembly pattern of hybrid **1** in: (**A**) CH_2_Cl_2_/heptane 1:1 7 mM; (**B**) CH_2_Cl_2_/heptane 2:8 7 mM; (**C**) CH_2_Cl_2_/EtOH 2:8 7 mM; (**D**) (HFIP)/EtOH 2:8 7 mM; and (**E**) DMSO/H_2_O 1:9 0.14 mM. Adapted with permission from [32]. Copyright 2019, Royal Society of Chemistry.

Coutsolelos and coworkers have recently reported an interesting study on the self-assembly process of two hybrids consisting of a peptide nucleic acid (PNA) linked with a tetraphenylporphyrin and boron-dipyrromethene (**1** and **2**, Figure 1a) [32]. Both hybrid subunits are well-known building blocks in the construction of self-organizing materials. In particular, PNAs result in excellent scaffolds for the construction of suprastructures involving different chromophores, thanks to their chemical structures that offer varied interaction modes such as stacking, hydrogen-bonding, and Watson-Crick base pairing. The aggregation of **1** and **2** was carried out in mixed solvent mixtures and the process was found strictly dependent on the solvent pair as well as their concentration. An obvious influence of the molecular structure of the hybrids (i.e., chromophore attached to the PNA moiety) was also evidenced. Eventually, the deposition method represents a further discriminating factor, dictating the final morphologies of the formed aggregates. Indeed, the drop casted solutions of hybrid **1** gave spheres with variable diameters depending on the solvent used, with the best result in term of size uniformity in the case of a 0.14 mM solution in DMSO/H_2_O 10% *v*:*v*, as evidenced by 

High Resolution Transmission Electron Microscopy (HRTEM) analysis (Figure 1b). This underlines that PNA molecules adopt the same spherical self-assembled structures in similar conditions, whereas the unfunctionalized 5-(4-aminophenyl)-10,15,20-triphenylporphyrin units did not to form specific nanostructures using the solvent driven aggregation methodology. As a consequence, the authors concluded that the PNA molecule is crucial for the assembly process, which is guided by the instauration of hydrogen bonds between the PNA units within the hybrids. However, the introduction of the chromophore was not ineffective, even if it was more relevant in the case of conjugate 2, where a different morphology (spherical or flake) was observed for the resulting assemblies in different solvent conditions.

UV–Vis and fluorescence spectroscopic studies have provided insights into the configuration of the porphyrin units within the assemblies. Using different solvent combinations (i.e., CH_2_Cl_2_/EtOH 2:8; CH_2_Cl_2_/heptane 1:1; 1,1,1,3,3,3,-hexafluoroisopropanol (HFIP)/EtOH 2:8; DMSO/H_2_O 1:9 *v:v*; and glass surface) as the deposition substrate, UV–Vis showed broadening and red-shifted bands in the porphyrin absorption region with respect to the corresponding bands of the hybrid system in monomeric form (a dichloromethane solution), indicating a J-type disposition of the macrocycles within the nanospheres. Fluorescence studies revealed an energy or electron transfer occurred between PNA and porphyrin units, with red-shifted emission bands for the film deposited of **1**, confirming the J-aggregate formation. Fluorescence microscopy experiments have shown that the conjugates are fluorescent in their self-assembled states, so their use in photovoltaic applications has been further investigated. The light-harvesting ability was verified for photocatalytic H_2_ production in the presence of Pt nanoparticles and ascorbic acid used as sacrificial agent. The hydrogen gas production activity was found to be 74.43 nM in 4.5 h, demonstrating the ability of the assembled nanostructures as light harvesting and templates for Pt photoreduction. 

**Figure 2:**
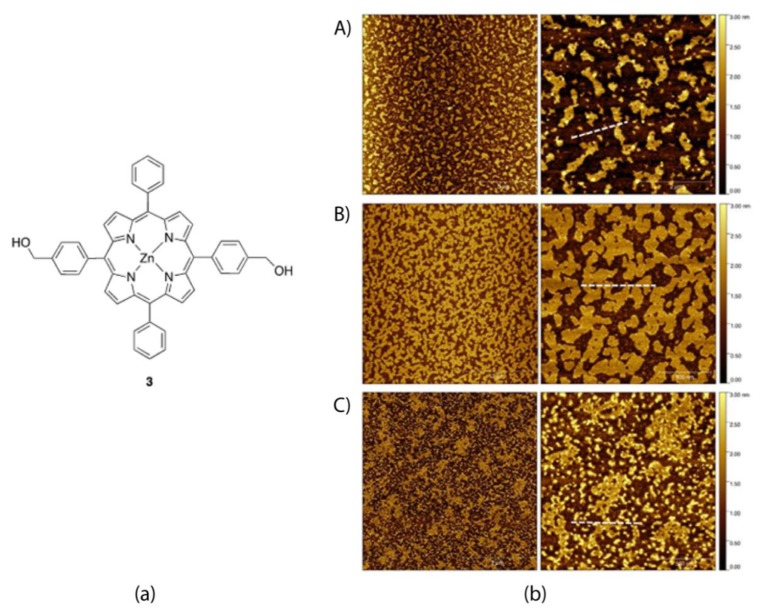
(**a**) Molecular structure of porphyrin **3** studied by Bonifazi and coworkers; (**b**) Atomic Force Microscopy images of samples prepared by spin coating a solution of **3** in: (**A**) CHCl_3_ scale bars 5 and 2 μm; (**B**) MCH (0.3% CHCl_3_), scale bars 5 and 0.5 μm; and (**C**) MCH (0.07% THF), scale bars 5 and 0.5 μm. Adapted from OPEN ACCESS [33].

Self-organization of porphyrin derivatives by hydrogen-bonding interactions has also been reported by Bonifazi and coworkers, who described the self-assembly behavior of a zinc porphyrin carrying two hydroxymethyl groups trans to each other in different solvent conditions [33]. Porphyrin **3** (Figure 2a) was efficiently prepared by a multi-step protocol, leading to the corresponding porphyrin di-ester, which was easily converted to the benzyl alcohol compound by reduction with LiAlH_4_. The following insertion of zinc ions in the porphyrin core yielded the desired product **3**, whose molecular packing in J-aggregated species have been obtained by x-ray spectroscopic analysis performed on crystals grown from the THF/cyclohexane solution. Regarding the self-assembly in the solution of **3**, it was found that the formation of weak aggregates occurred when porphyrin was dissolved in pure chloroform, but the use of a more apolar solvent mixture, namely methylcyclohexane/chloroform (99.7/0.3, *v*/*v*), favored the formation of stronger supramolecular assemblies. In both cases, the monomeric form can be restored by adding pyridine or methanol that disrupt both axial metal coordination and hydrogen-bonding interactions, respectively, which are responsible for the assembling process. Similar results have been obtained by performing the aggregation in a methylcyclohexane/THF solution with 0.07% THF content. Atomic Force Microscopy (AFM) studies have shed light on the morphology of the aggregates obtained by different solvents and spin-coated on a mica surface, confirming the results collected by spectroscopic studies. In general, two-dimensional islands of 2 nm in height were observed, but the size of the aggregated structures and their homogeneity were strictly dependent on the solvent used (Figure 2b). Indeed, the observed architectures varied from the smaller and scattered islands obtained in the chloroform solution and the more extended 2D systems of the mixed solution methylcyclohexane(MCH)/chloroform up to the heterogeneous structures of variable size observable for the MCH/THF solution. In the last case, the use of a tiny amount of THF partially disaggregated the stacked systems, competing with the hydroxyl groups for both hydrogen-bonding and Zn complexation. 

**Figure 3:**
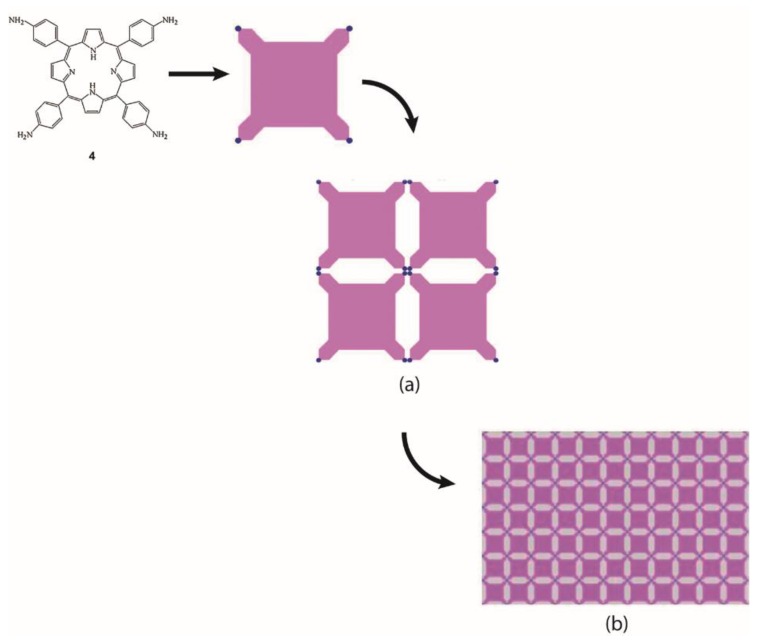
Schematic representation of the formation of nanorods and nanothorns based on porphyrin **4** as reported by Yun and coworkers. (**a**) formation of the tetrameric supramolecular structure of the self-assembled nanorods and nanothorns; (**b**) formation of ordered porphyrin structures by its three-dimensional expansion. Adapted with permission from [34]. Copyright 2014, Elsevier.

Hydrogen-bonding is crucial to achieve the ordered arrangement of meso-tetrakis(4-aminophenyl)porphyrin **4** (Figure 3a) in different nanostructures as reported by Yin et al., who generated nanospheres, nanorods, and nanothorns by modifying porphyrin concentration and using the phase-transfer method [34]. Typically, assemblies were made by rapidly adding 1 mL of methanol to 4 mL of the chloroform porphyrin solutions at different concentrations ranging from 5 × 10^−^^4^ to 1.5 × 10^−^^3^ M, and stored for three days to have stable nanosystems for morphological analyses by Transmission and Scanning Electron Microscopies (TEM and SEM, respectively). Remarkably, the authors were able to directly image the formation of a quadrate unit of fringes held together thanks to the hydrogen-bonding between the amino groups by HRTEM in both nanorods and nanothorns. UV–Vis spectroscopic analysis revealed a broadened, red-shift of the Soret band for the nanoaggregates of ca. 20 nm with respect to that of the porphyrin in the monomeric form, indicating that the porphyrins were aggregated in J-type systems within the nanoaggregates. 

Based on the collected data, the formation of the ordered arrays of porphyrin **4** can be depicted as shown in Figure 2: first, a tetrameric supramolecular structure of the self-assembled nanorods and nanothorns is formed, thanks to both hydrogen-bonding and π–π interactions (Figure 3a), then its three-dimensional expansion is brought to the formation of highly ordered arrangements of porphyrin molecules on a large scale (Figure 3b).

It is well-known that the electronic delocalization in J-type porphyrin aggregates confers semiconducting properties to the assemblies, which can be used as photocatalysts to harvest light and generate electron-hole pairs if irradiated. In this regard, Bhosale and coworkers very recently produced ordered porphyrin aggregates of 5,10,15,20-tetrakis(pentafluorophenyl)porphyrin in THF/H_2_O solvent mixtures and investigated their use for the photodegradation of rhodamine (Rhb) under visible light irradiation [35]. Porphyrin monomers, whose hydrophobic character was enhanced by the introduction of several fluorine atoms on the phenyl rings, self-organized in aggregated structures when 70 and 80% of water was added to the THF solution. At these percentages, the macrocycles formed J-aggregates by π–π stacking interactions as shown by the red-shift and broadening of the Soret band, quenching of the fluorescent emission intensity, and the shift in the stretching vibrations of C=C groups from 1474 to 1493 cm^−^^1^, as indicated in the Fourier Transform Infrared (FT-IR) spectrum. Interestingly, SEM images showed that the morphology of the aggregated microstructures was deeply influenced by the % H_2_O proportion: indeed, microrods with diameters of 1–3 μm and lengths in the range of 20–100 μm were characteristic of the aggregates obtained in the 3/7 THF-H_2_O solution, while octahedral crystals of about 30 μm in size were formed at 80% of water in THF. The different morphologies displayed by the two materials also influenced their photocatalytic ability in degrading RhB molecules under simulated sunlight irradiation, which was higher for the microrods with respect to the crystals (with rate constants measured of 3.76 × 10^−3^ and 2.93 × 10^−3^ min^−1^, respectively), as a consequence of their higher surface area available for the photooxidation process. Furthermore, the assemblies showed higher photocatalytic activities with respect to the amorphous porphyrins, since a better charge separation than that of monomers was achieved for the ordered aggregated species.

**Figure 4:**
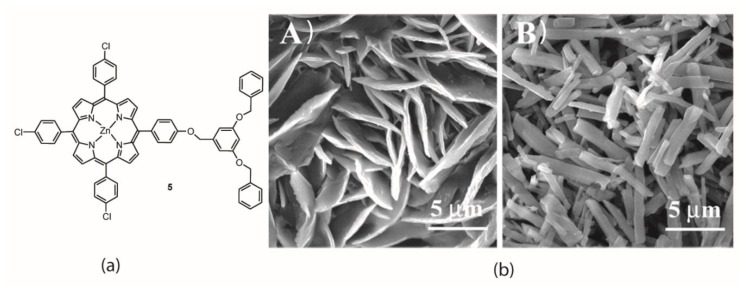
(**a**) Molecular structure of Zn porphyrin complex **5** studied by Zhu and coworkers [19]. (**b**) Scanning Electron Microscopy images of the aggregated structures formed in (**A**) *n*-hexane and (**B**) methanol. Adapted with permission from [36]. Copyright 2015, Elsevier.

For the Zn porphyrin complex **5** reported in Figure 4a, Zhu and coworkers described the different self-organization in twisted nanorods approximately 500–800 nm in width, 4–8 μm in length, and 500–700 nm in height, and micrometer-sized leaves of about 50 nm, in solvents of different polarity (i.e., methanol and n-hexane, respectively) (Figure 4b) [36]. UV–Vis studies indicated that, in the first case, macrocycles adopted the H-type aggregation mode, while in the apolar medium, J-aggregates are formed. These latter species are derived from starting dimeric structures held together mainly by π–π interactions between the phenyl groups of close porphyrins that eventually self-assembled in the J-type nanostructures. Conversely, in methanol, complementary hydrogen-bonding between the –OH groups of the solvent with oxygens of the porphyrin formed a weak **5**/methanol complex that altered the π–π interaction between the phenyl moieties of neighboring porphyrin molecules and made π–π interactions between neighboring porphyrin rings prevail. Twisted nanorods were formed by the intermolecular π–π interaction between porphyrin rings.

**Figure 5:**
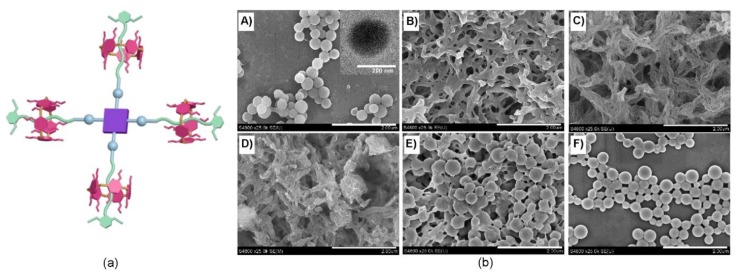
(**a**) Cartoon representation of the porphyrin-[5]-rotaxane system reported by Yang et al. (**b**) SEM images of the aggregated structures formed in a mixture of (**A**) CH_2_Cl_2_/*n*-hexane (1:9, *v*/*v*); (**B**) DCM/acetone (1:9, *v*/*v*); in CH_2_Cl_2_/acetone (1:9, *v*/*v*) after aging for (**C**) 0 h; (**D**) 12 days; (**E**) 16 days; and (**F**) 40 days. Scale bars: 2.0 μM. Adapted with permission from [37]. Copyright 2019, Elsevier.

Similar results have been reported by Yang and coworkers regarding the self-assembly behavior in different solvents of symmetric [5] rotaxane with Zn porphyrin as the core, platinum-acetylide as the linkage, and four arms decorated with pillararene (Figure 5a) [37]. When this system was dissolved in dichloromethane, it self-assembled in J-type aggregates by π–π interactions, as demonstrated by UV–Vis and emission spectroscopic investigations. The corresponding film casted onto a quartz substrate featured both Soret and Q bands slightly red-shifted and significantly broadened in comparison with that in CH_2_Cl_2_. The use of different solvent mixtures for the preparation of the aggregates gave varied nanostructures, whose morphologies have been characterized by SEM and TEM microscopies (Figure 5b). When the [5] rotaxane was exposed to a non-polar solvent (CH_2_Cl_2_: *n*-hexane = 1: 9, *v*/*v*), followed by slow evaporation in air at room temperature, it exhibited large-scale, solid spheres with an average size of 320 nm. The use of CH_2_Cl_2_:acetone = 1:9, *v*/*v* solution made the morphology of the [5] rotaxane change into a uniform and highly ordered fibrous network structure with a width of ca. 80 nm: the larger solvent polarity caused the stretching of the oligomers that were organized in nanofibers. Interestingly, in this case, the morphology evolved in half a month from tangles of nanofibers to nanospheres.

New insights on the different electrochemiluminescence (ECL) properties of H- and J-aggregates formed by the zinc complexes of 5,10,15,20-tetraphenylporphyrin (Zn TPP) molecules were recently reported by Lu et al. [38]. First, they prepared different types of aggregates using the mixed solvent method by adding methanol or cyclohexane to a chloroform solution of the porphyrin complex in proper proportion. In particular, H-aggregates with a size of 60–100 nm were obtained when the CHCl_3_/cyclohexane ratio was 1:19, while the J-type assemblies were achieved in CHCl_3_/CH_3_OH 19:1 *v/v* as elongated rods of several micrometers in length and 2 nm in height. Fluorescence studies revealed that J aggregates possessed better fluorescence properties than the H-type in terms of both intensity and quantum yield, which were found to be more than ten times and ca. 5% higher than the face-to-face suprastructures, respectively. The evaluation of ECL properties was carried out by electrochemical studies with the prepared aggregates deposited onto a glassy carbon electrode (GCE) surface as 5 μM solutions in 0.1 M PBS (pH = 7.4) and in the presence of 0.04 M K_2_S_2_O_8_. The authors described the ECL emitting mechanism for both the aggregates in four steps, as outlined in the following equations:

ZnTPP-Agg + e^−^ → ZnTPP-Agg^●−^ (1)

S_2_O_8_^2−^ + e^−^ → SO_4_^2−^ + SO_4_^●−^ (2)

ZnTPP-Agg^●−^ + SO_4_^●−^ → SO_4_^2−^ + ZnTPP-Agg* (3)

ZnTPP-Agg* → ZnTPP-Agg + hν(4)

In brief, the initial reduction of aggregated species on GCE surface (Equation (1)) produces the radical anion form ZnTPP-Agg^●−^, which reacts with the radical SO_4_^●−^ produced by the reduction of the S_2_O_8_^2−^ ion (Equation (2)) to give the excited state ZnTPP-Agg* (Equation (3)) that loses energy in the form of light, coming back to the ground state (Equation (4)).

The ECL intensity of the J-aggregates was found to be one order of magnitude higher than that of H-aggregates; moreover, the reduction peak of −1.384 V in J-aggregates was lower than that of H-aggregates. This was reasonably explained by the narrow band gap between HOMO and LUMO orbitals characterizing J-aggregates (red-shifted absorptions), and this fact not only benefits the electron-hole recombination, but also facilitates the electron injection into J-aggregates, favoring the progress of Equation (1). Furthermore, the higher number of Zn porphyrin monomers in a unit J-aggregate with respect to the corresponding H-aggregate unit enhances the possibility of generating excited states, with obvious repercussions on the strength of the ECL intensity.

**Figure 6:**
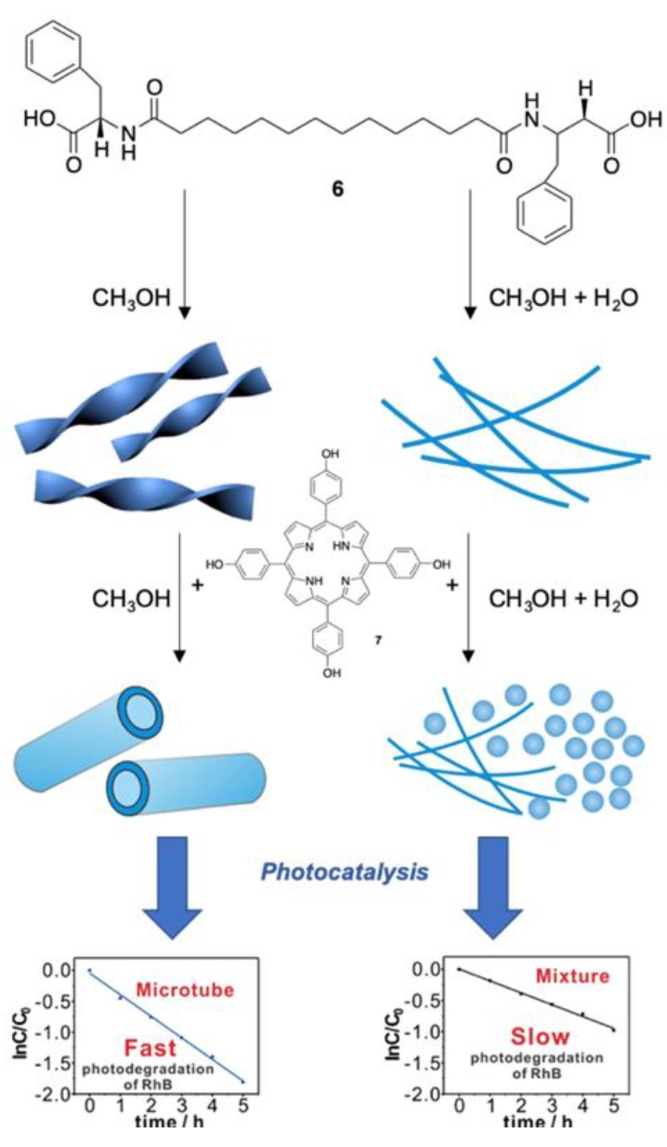
Schematic illustration of the Bola-F/THPP systems obtained by Jiang et al. with morphologies tunable by the solvent used (i.e., methanol and methanol/water mixture). Adapted from OPEN ACCESS [39].

Jiang et al. have recently reported on the construction of molecular assemblies using bolaamphiphilic molecules containing a medium length alkyl chain and l-phenylalanine head groups (Bola-F, **6**) and tetrakis-5,10,15,20-(4-hydroxyphenyl)porphyrin **7** [39]. The nanostructures obtained displayed distinctive morphologies depending on the solvent used, with remarkable repercussions on their photocatalytic properties (Figure 6). In detail, when dissolved in methanol, Bola-F molecules self-assemble in microscale twists resulting from the chirality of the aminoacidic residues and held together by a combination of hydrogen-bonding and hydrophobic interactions. When porphyrin **7** is added to the methanolic solution of **6** (molar ratio of 1/250), the resulting co-assemblies show (in SEM images) microtubular structures where porphyrin units are strongly aggregated in J-type species, as evidenced by the UV–Vis spectral absorptions where the Soret band is remarkably red-shifted to 478 nm. However, when co-assembly is carried out in MeOH/H_2_O (1:1, *v*/*v*), in a molar ratio of 5/9 of **6** and **7**, both fibers and small spherical nanoparticles with a diameter of ca. 15–300 nm were obtained by fast precipitation. In addition, in this case, porphyrin J-aggregation was induced by bolamphiphiles, as suggested by the red-shift of the Soret band to 475 nm. This underlines that for the same weight of the co-assemblies obtained using different solvents, that assembled in MeOH/H_2_O contained significantly more porphyrin units in comparison with that of the microtubular ones. The investigation of photocatalytic properties shows that Bola-F/**7** fibers/nanospheres photodegrade about 60% of RhB molecules under irradiation, whereas the microtubes reach more than 90% of conversion within 5 h. This contrasts with the porphyrin content in the microtubes, which is only 0.22% of that of the fibers/spheres mixtures. The authors explain this unexpected result by stating that the large extent of porphyrin aggregation within the fibers/nanospheres could have a consistent energy transfer that hampers the electron transfer necessary to produce the reactive oxygen species.

**Figure 7:**
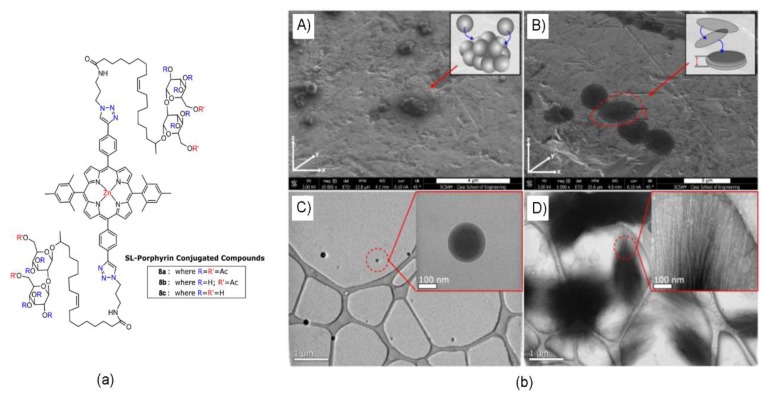
(**a**) Molecular structure of sophorolipid-Znporphyrin compounds investigated by Singer t al. (**b**) SEM images of (**A**) **8a** and (**B**) **8c** taken from a 45° tilt angle and TEM images of (**C**) **8a** and (**D**) **8c**. Reprinted with permission from [40]. Copyright 2019, American Chemical Society.

The self-aggregation of a series of glycolipid-functionalized zinc porphyrin complexes in dilute MeOH/H_2_O (1:1 by volume) solution was recently investigated by Singer et al. [40]. They prepared the three porphyrin derivatives reported in Figure 7a, where two sophorolipid units (SL) with a variable number of acetyl groups are located at the 5- and 15- meso-phenyl peripheral positions. The different hydrogen-bonding ability of the peracetylated (**8a**), diacetylated (**8b**), and nonacetylated (**8c**) compounds strictly influences their self-assembly behavior, leading to aggregated species of different morphologies and chiral features conferred by the carbohydrate moieties.

Although a J-type aggregation was recognized by UV–Vis spectroscopic analysis for all the derivatives investigated, the presence of a clearly resolvable split-Soret band for compounds **8b** and **8c** was indicative of strong interacting macrocycles, furthermore displayed by the intense trisignate (−/+/−) CD bands in the 423–447 nm region, demonstrating that the left-handed helical aggregation was driven by the hydrogen-bonding between properly conformed carbohydrate units whose stereogenic information was read during the spatial macrocycle organization. Porphyrin **8a** weakly aggregated in specific systems not characterized by supramolecular chirality, since it lacks hydroxyl groups. The SEM and TEM microscopy provided more detailed information on the 3D nature of the obtained aggregates. The fully acetylated porphyrin compound **8a** self-assembled into submicron spheres, while the other two derivatives formed elongated fibrillar structures, laterally stacked in a hierarchical fashion (Figure 7b). Investigations aimed to elucidate the self-assembly mechanism have been performed by monitoring the temperature-sensitive excitonic-band evolution for **8b** and **8c**, and the low-energy shoulder of the UV–Vis band at 439 nm for derivative **8a**, whose aggregates are CD silent. From the cumulative results collected, an isodesmic (noncooperative) assembling process was observed for compound **8a**, with the initial formation of micellar aggregates that evolved into spherical structures of increasing dimensions by polymerization. In contrast, aggregation of porphyrins **8b** and **8c** proceeds by a cooperative (nucleation-elongation) growth pathway, which is particularly favored for compound **8c**, where the higher number of hydrogen-bonding interaction sites aids in the cooperative nature of association.

**Scheme 1:**
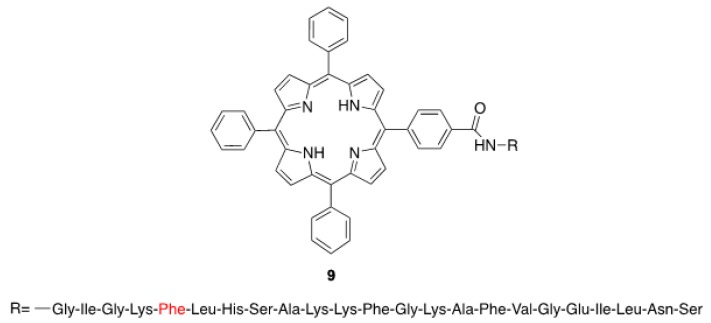
Molecular structure of TPP-(l)-magainin 9 investigated by Gobbo et al. [41].

Gobbo and Monti’s groups have investigated the solvent-promoted formation of chiral assemblies based on porphyrin-aminoacid(s) conjugates by the hydrophobic effect, where several structural parameters were varied in order to study their influence on the supramolecular chirality displayed by the final assemblies. As a part of their studies devoted to the development of porphyrin photosensitizers, Gobbo et al. [41] have prepared many porphyrin-peptide conjugates, noting that the derivative TPP-(l)-magainin **9** (Scheme 1) formed large chiral suprastructures with α-helical folding of the peptide chain. To assess the role of both the screw-sense of the peptide helix and the linker sequence in determining the chiral features of the aggregates formed in MeOH/H_2_O media, the authors prepared a series of synthetic porphyrin derivatives linked to different α-helical peptides. Circular Dichroism (CD) spectroscopic studies, supported by preliminary molecular dynamic simulations, revealed that the chiral arrangement of porphyrin derivatives was strictly determined by the linker between porphyrin and the peptide chain, rather than of the coiling of helices. Moreover, the introduction of a chiral α-aminoacid within the peptide sequence allowed for tuning the morphology of the obtained assemblies by the inversion of the configuration of the stereogenic center.

**Figure 8:**
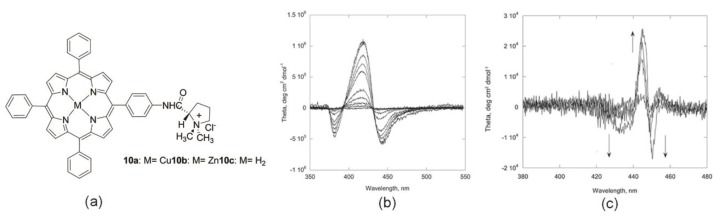
(**a**) Molecular structure of the conjugated (L)-proline-porphyrin complexes studied by Monti et al. (**b**) CD variation with time of **10a** in EtOH/H_2_O (30%, *v*:*v*) 2.8 × 10^−6^ M. (**c**) CD spectral variation with time of **10b** in EtOH/H_2_O (25%, *v*:*v*) 4.0 × 10^−6^ M. Adapted with permission from [42]. Copyright 2019, Royal Society of Chemistry.

Very recently, Monti et al. reported the possibility of forming chiral suprastructures starting from Zn and Cu cationic (l)-prolinate-tetraarylporphyrin complexes in different aqueous media (Figure 8a) [42]. As reported by the authors in previous works, the appended-proline group provides the chiral information to read during the aggregation process, and directly determines the chiral features of the formed assemblies, together with other boundary experimental parameters like solvent composition, monomer concentration, charge of the proline unit, and the metal ion coordinated. The use of dimethylacetamide/water solvent mixtures did not allow the formation of specific, chiral aggregates for the two derivatives. Indeed, the copper derivative **10a** self-assembled in aspecific structures by a cooperative mechanism, featuring coalescing dichroic bands during the aggregation. Similarly, the Zn complex gave aggregates in aspecific mode, CD silent, and with macrocycles scarcely interacting, as evidenced by the resonance light scattering measurements. In EtOH/H_2_O, the Cu porphyrin monomer **10a** self-organized in J-type aggregates as indicated by the evolution of the absorption peak centered at 444 nm. The CD spectral profile showed clear bisignate, negative dichroic bands, suggesting a counter-clockwise arrangement between the chromophores (Figure 8b). The higher intensity of about two orders of magnitude of the CD signal with respect to the assembling in dimethylacetamide/water medium was ascribable to the lower ability of EtOH to solvate the highly polarizable porphyrin platforms, favoring a closer contact of the macrocycles in the aggregated species. The self-assembly of the complex **10b** in 25% aqueous ethanol led to the formation of J-aggregates by the reaction limited aggregation (RLA) mechanism, featuring s CD spectral pattern composed of two dichroic bands centered at 450 and 430 nm, illustrating porphyrin architectures with a rod-like morphology (Figure 8c). Dynamic light scattering studies (DLS) in EtOH/H_2_O solutions revealed an average diameter of 90 nm for the copper complex, while a more complex behavior was obtained by the Zn(II) counterpart. In fact, two populations with different sizes have been detected (90 nm × 1 μm) evolving with time, and are representative of asymmetric-elongated rod-type structures, in accordance with the spectral pattern of CD spectra above-mentioned.

**Figure 9:**
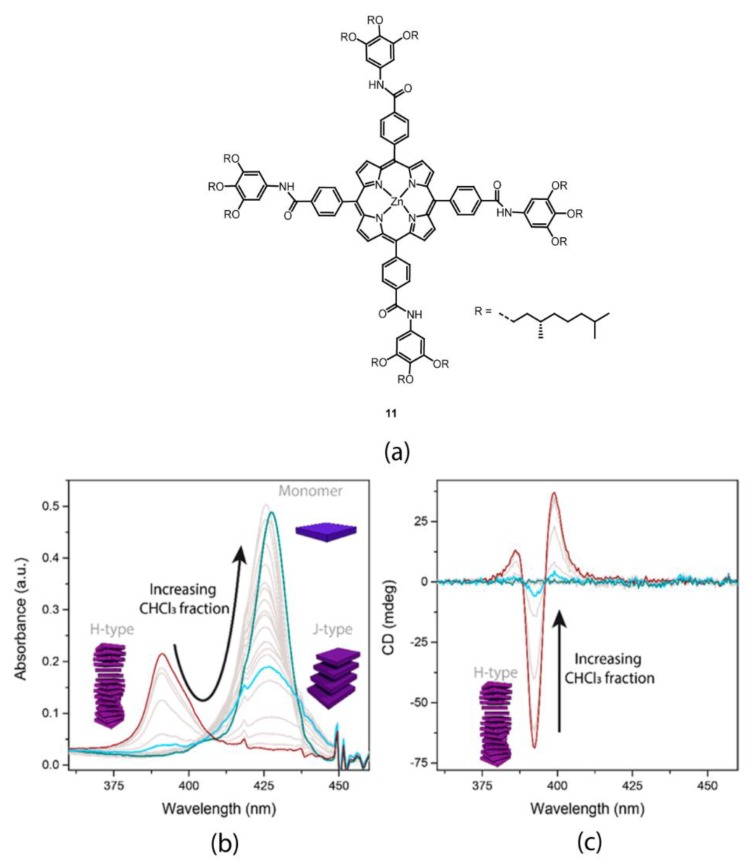
(**a**) Molecular structure of chiral amide-functionalized Zn porphyrin **11** investigated by Meijer et al. Solvent dependent (**b**) UV–Vis and (**c**) CD spectra of 1 μM solutions of **11**, going from methylcyclohexane to solutions with increasing proportions of chloroform. Adapted with permission from [43]. Copyright 2018, American Chemical Society.

Meijer et al. recently reported an interesting study on the pathway complexity featured by the amide-functionalized porphyrin **11** (Figure 9a) where self-aggregation was performed at various concentrations, solvent compositions (MCH and chloroform mixtures), and temperatures [43]. The authors found that when porphyrin **11** was dissolved in pure MCH, chiral H-aggregates were formed, with the macrocycles co-facially stacked and held together by hydrogen-bonding, as clearly indicated by the absorption band centered at 392 nm, displaying a strong cotton effect in the CD spectrum (Figure 9b,c). The addition of the increasing CHCl_3_ fraction resulted in a progressive lowering of both the H-aggregates absorption and dichroic bands, concomitant with the emerging of a new, CD silent absorption band at 425 nm, ascribable to the porphyrin organization in weakly aggregated J-type species. When 5–7.5 vol% CHCl_3_ was reached in solution, H-aggregates were completely disrupted in favor of J-type assemblies, whose stability was affected by the further addition of chloroform, which led to disaggregation into monomeric forms. The formation of two different aggregate types was also confirmed by FT-IR spectroscopy, using the NH stretch and amide CO stretch frequencies as diagnostic peaks. The employment of the thermodynamic model to fit the experimental data allowed the authors to determine the two-pathway polymerization process and its dependency by solvent, concentration, and temperature conditions. It was found that higher concentration conditions increased cooperativity in the system. Furthermore, the thermodynamic model revealed that the competition between the cooperative and isodesmic aggregation pathways resulted in an increase in both the solvent composition and temperature values at which H-aggregates can form. In contrast, this type of aggregate cannot organize at chloroform fractions or temperatures above the critical fraction or elongation temperature, as a consequence of the buffering effect of the isodesmic J-type aggregation, which was remarkably responsive to solvent composition and temperature, but not to concentration variations.

The self-assembly of large organic π-conjugated molecules with disc-like shapes has been investigated for a long time given their potential in creating nanomaterials to employ in energy or charge transport in electronics. Among them, the C_3_ symmetrical structures are the most popular, where the central core is functionalized with three identical arms that drive the supramolecular organization, determining the morphological features at different length scales. In particular, suprastructures derived by the assembly of monomers possessing 1,3,5-benzenetricarboxamides (BTA) as the central core, have been extensively studied, since this central unit can be directly functionalized by an amide peptide bond or by introducing a spacer like 3,3′-diamino-2,2′-bipyrdine (Bipy), which controls the self-assembling behavior of the monomer. In this regard, herein we report on two recent examples of discotic molecules, whose aggregation mode and final morphology is strictly dependent on the solvent.

**Figure 10:**
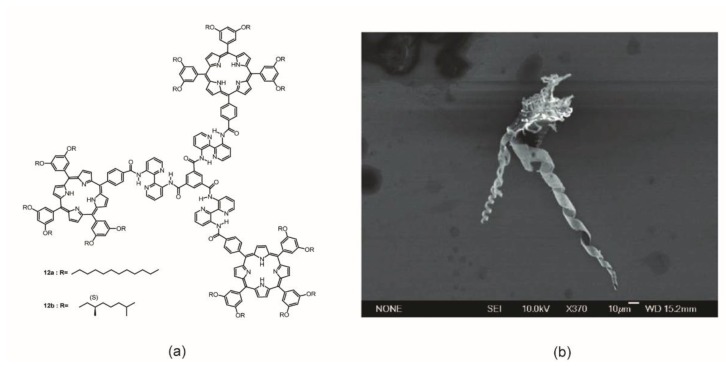
(**a**) Molecular structure of achiral and chiral BTA–porphyrin discotic molecules studied by Martín et al. (**b**) SEM image showing the superhelix obtained by self-assembly of **12b** in heptane (1 mg/mL, scale bar 10 μM). Adapted with permission from [44]. Copyright 2017, Royal Society of Chemistry.

Martín et al. reported on the preparation and self-assembly behavior of two elaborated porphyrin trimeric architectures possessing BTA–Bipy cores **12a**,**b** (Figure 10a) [44]. The basic idea was that porphyrin trimers and BTA–Bipy discotic molecules would arrange in triple helical bundles more extended than that just reported by others. During SEM morphological studies performed on aggregates formed by the chiral derivative **12b** in heptane, the authors serendipitously discovered a μm-sized helical structure between many other undefined structures (Figure 10b).

The investigation of the self-assembling process at diluted concentrations by a combination of several spectroscopic techniques (^1^H-NMR, UV–Vis, CD, fluorescence) allowed for the identification of the interactions that drive the arrangement of the porphyrin–BTA–Bipy systems. In detail, the Bipy moiety was found to have a great impact on the monomer structure, since it favored the establishment of intramolecular hydrogen bonds between the aromatic nitrogen atoms and the N–H of the amides, thus forcing a flat conformation for either the entire molecule or just the 2,2′-bypiridine unit. When monomers were dissolved in n-heptane or methylcyclohexane, at μM concentrations, the formation of suprastructures held together by π–π stacking interactions was observed, whose chiral features were also evident for the chiral monomer **12b**. Fluorescence measurements in heptane further clarified the intermolecular/energetic communication between Bipy and porphyrin in solution, witnessed by the lack of the typical strong green emission at 520 nm of the stacked coplanar Bipy units. TC-CD experiments revealed the formed assemblies as highly thermodynamically stable structures, sensitive to solvent quality since the addition of chloroform or 1,1,2,2-tetrachloroethylene disrupted the architectures. Finally, “Sergeant-and-Soldiers” experiments by mixing achiral and chiral monomers in n-heptane, at a μM concentration, showed no amplification of chirality, indicating that the monomer exchange was not thermodynamically favorable. The formation of aggregates did not seem to display thermodynamic reversibility.

**Scheme 2:**
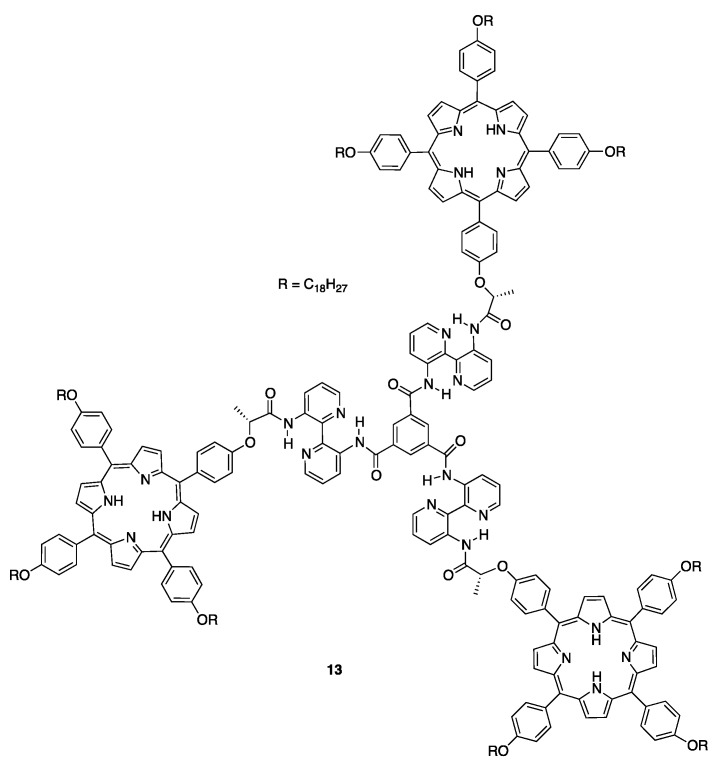
Molecular structure of C_3_ symmetric tris-porphyrin discotic molecule **13** studied by Avarvari et al. [45].

More recently, similar porphyrin disc-like architectures possessing a BTA–Bipy core was synthesized by Avarvari and coworkers and its self-assembly behavior was studied in solvents of different quality, namely chloroform, 1,4-dioxane, methylcyclohexane, and dodecane, in order of decreasing polarity (Scheme 2) [45]. The main difference with respect to the system described by Martín et al. consisted of the presence of a chiral spacer between the rigid core and the three porphyrin platforms that strongly affected the packing mode of the monomers in the final aggregates, as discussed below. CD spectroscopy revealed that monomers **13** weakly assembled in the more polar solvents (i.e., chloroform and 1,4-dioxane), giving a bisignate spectral feature (−/+) of low intensity. In contrast, the self-assembly in apolar solvents gave chiral structures featuring strong trisignate signals, slightly red-shifted and one order of magnitude higher than in the more polar ones, indicating the occurrence of J-type aggregation. Despite the usual aggregation mode observed for the families of C_3_ molecules based on BTA–Bipy cores determined by π–π stacking interactions of these units, for such molecules the aggregates were formed through a close interaction between the porphyrin macrocycles, as suggested by the CD active Soret band. Theoretical calculations on simpler dimeric and trimeric species indicated that the methyl group of the stereogenic center in compound **12** deeply affected the molecular conformations, preferring to sit on the axial position, with the phenyl group of the porphyrin located below the core of the molecule. These peculiar structures were found to be variously favored by solvent quality in terms of noncovalent interactions, tending to aggregate in apolar solvents and to remain quite isolated in polar solvents. This allowed the authors to warrant the large CD signal recorded in the first case to the intermolecular interactions between porphyrins of close monomers, and the weak CD signal was a result of intramolecular chirality between porphyrin within the same monomer in the latter. AFM measurements showed that only globular structures of different sizes, depending on the solvent used, were formed, in contrast with the fibrillar one dimensional aggregates generally observed for other C_3_ discotic molecules, probably due to the nondirectional interlocking of porphyrin rings determined by the chiral spacer.

**Figure 11:**
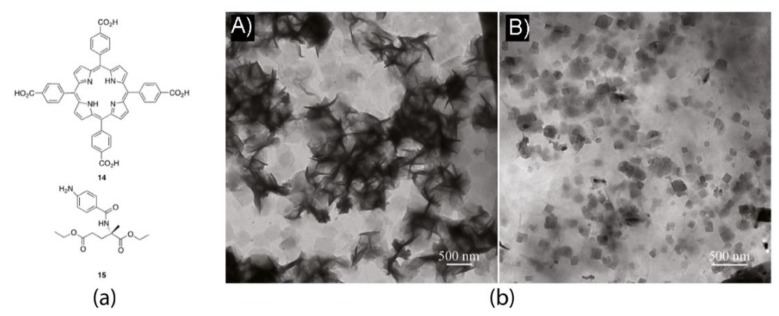
(**a**) Molecular structure of porphyrin **14** and l-glutamic acid diethyl ester **15** studied by Peng et al. (**b**) TEM images of **14**/**15** at the volume ratio of (**A**) DMF/CHCl_3_ at 2/8 and (**B**) DMF/H_2_O at 3/7. Reprinted with permission from [46]. Copyright 2019, American Chemical Society.

Very recently, Peng et al. prepared composite film nanostructures starting from the achiral tetrakis-5,10,15,20-(4-carboxyphenyl)porphyrin **14** and l-glutamic acid diethyl ester **15** (Figure 11a), whose morphologies were found to be remarkably different with the DMF/H_2_O or DMF/CHCl_3_ solvent mixtures used to induce the aggregation process [46]. Furthermore, the presence of a stereogenic center on the l-glutamide derivative determined the supramolecular chiral character of the aggregated species, even if a direct correlation between the chirality of the used molecules and the obtained nanostructures was not found. Morphological analyses by SEM and TEM microscopy showed that by simply modifying the solvent conditions (combined volumes and solvent type), different nanostructures were obtained: the definitive solvent compositions used by the authors were DMF/CHCl_3_ (2:8, *v*/*v*) and DMF/H_2_O (3:7, *v*/*v*), which determined the flower-like and brick-like structures in the corresponding nanocomposite films, respectively (Figure 11b). In addition, a crucial concentration value of 2 mM of **15** was found to homogeneously form the above-mentioned structures. UV–Vis and fluorescence studies on the cast films gave more information on the porphyrin **14** arrangements in the two distinctive nanostructures. In the DMF/H_2_O solvent mixtures, a blue-shifted Soret band of ca. 12 nm was observed for the cast film in comparison with the analogous film based on the monomeric, not aggregated **14** form in DMF, implying the formation of co-assemblies via the H-type aggregation mode. On the contrary, films obtained in DMF/CHCl_3_ showed a red-shifted Soret band of ca. 10 nm, indicative of the J-type mutual arrangement of porphyrin macrocycles. This opposite feature is also confirmed by CD spectroscopy, where an intense CD split band located at 446 nm with a positive maxima CD signal at 452 nm and a minimum negative band at 433 nm was observed for assemblies grown in DMF/CHCl_3_. In the case of the DMF/H_2_O system, the cast film showed an intensive negative CD band at 450 nm and a positive band at 437 nm, with a crossover at 440 nm. More importantly, in comparison with the **14**/**15** system in DMF/H_2_O, the Cotton effect of **14** with the assemblies in **15** in DMF/CHCl_3_ solvents was slightly red shifted, which was in accordance with data of the UV−Vis spectra. These differences are likely to be induced by different solvents and the chiral sense of **15** effectively transferred to the porphyrin chromophores, which was steered by hydrogen-bonding between the two subunits. At the same time, it also indicates that the different orientations in the molecular packing caused the reversed supramolecular chirality. Interestingly, the authors investigated the enantioselective ability of the mixed films toward different chiral amino acid solutions by water contact angle measurements. Indeed, because of the differences in the packing nanostructures within the composite films, the interaction with the amino acid solution alters the contact angles which was found to be 116.6° and 99.1° for the deposited films produced by DMF/CHCl_3_ (2:8, *v*/*v*), and DMF/H_2_O (3:7, *v*/*v*), respectively, and their change depends on the hydrophobic/hydrophilic character of the surface-containing nanostructures.

## 3. Effects of Chemical External Stimuli

It is well-established that the largest part of porphyrin assemblies is organized in aqueous media, since several solution parameters can be changed to prompt the aggregation in a controlled manner like pH and ionic strength (added salts). Most of these works exploited water soluble porphyrins such as 5,10,15,20-tetrapyridylporphyrin (TPyP) or 5,10,15,20-tetrakis(4-sulfonatophenyl)porphyrin (TPPS), with the latter as the undisputed protagonist. Furthermore, the addition of organic or inorganic species acting as templates in aqueous solutions can induce the organization of versatile and functional structures of practical interest. In this section, recent examples dealing with the above-mentioned approaches are reviewed.

### 3.1. pH and Added Salts

The pH of the media may have a profound influence on the aggregation state of the tetrapyrrolic macrocycles. In strongly acidic media (pH~2), for example, the nitrogen-core atoms can be easily protonated, causing a change in the charge state of the macrocycles with a distortion of the planar geometry toward a saddled structure. These two factors may act in different ways, depending on the structure of the porphyrin. In the presence of anionic substituents, a favorable electrostatic interaction could be onset, promoting the self-assembly of the obtained zwitterionic species [47]. On the contrary, coulombic repulsions would prevail, hampering the formation of supramolecular structures. This latter effect is often more pronounced in more polar media, due to the stabilization of the monomers either by specific (hydrogen-bonding interaction) or by general solvation (polarity effect) [48].

This was found by Zannotti et al. during the studies of the solvent-promoted aggregation of the tetrakis-5,10,15,20-(4-hydroxyphenyl)porphyrin **7**, which occurs at neutral pH, going from pure ethanol to a water prevalent solvent mixture [49]. The aggregation, steered by the hydrophobic effect, results in both H- (face-to-face) and J-structures (offset side-to-side) [50], as demonstrated by spectroscopic techniques and molecular dynamic simulations. At acidic pH, the predominant forms are the monomeric species, due to electrostatic repulsion between the di-cationic protonated rings and hydrogen-bonding interaction with the solvent.

As formerly introduced, in the case of negatively charged macrocycles, the protonation of the central nitrogen core is essential for steering the self-assembly process. In the case of the well-known tetrasulfonatophenyl derivatives TPPS, the aggregation is carried out at very low pH, in the presence of strong acidic species. Interestingly, a peculiar effect of the nature of the counterion on both the kinetic and the extent of aggregation has been found by Monsù Scolaro et al. [51]. The growth of the aggregates was promoted in the order H_2_SO_4_ > HCl > HNO_3_ > HClO_4_, indicating a correlation of the effect on the structure-making or breaking properties (i.e., cosmotropic or chaotropic ions, according to Hofmeister series) [52] of the different anions with respect to the hydrogen-bonding network of the solvent. Very interestingly, the same effects have been found to affect the chirality of the final species, arising from a scalemic symmetry breaking event during the self-recognition steps. Although, in principle, supramolecular species constituted by achiral molecular building blocks should not feature asymmetric properties, assemblies made of TPPS protonated zwitterionic species are intrinsically chiral due to the tacticity of the condensed forms [53,54]. However, this should result in the statistical observation of either positive and or negative CDs (arising from randomly prevailing scalemic mixtures) as well as silent spectral patterns (formation of racemic mixtures), where several published results have pointed out the prevalence of spectral patterns with positive bands (i.e., arising from a clockwise mutual disposition of the porphyrin macrocycles). The nature of this phenomenon is far from being fully explained, and is still under debate [55,56,57].

However, the stereoselective effect of chiral co-solutes was found by the same authors on the aggregation behavior of porphyrin-based ionic liquids in acidic chlorinated solvents [58], as demonstrated by the fact that in the presence of a specific enantiomer of added R- or S-camphorsulfonic acid (CSA) acting as a chiral template, CD spectral features appear, with specific positive or negative signs depending on the selected CSA. The effect of the nature of alkali metal cations on the aggregation of the paradigmatic TPPS has been studied by Leishman and McHale [59], who pointed out differences in the morphology of the aggregates in the presence of MCl species (M = H^+^; Li^+^; Na^+^; K^+^; and Cs^+^). Spectroscopic studies based on electronic absorption, resonance light scattering, and resonance Raman spectroscopy as well as imaging data, revealed a complex nanotubular morphology of the aggregates, whose intimate structures are environment-dependent. Cosmotropic, strongly solvated species cause a stiffening of the supramolecular structures, whereas weakly bound chaotropic cations promote an extensive solvation of the aggregate structures, associated with a larger local and orientational disorder.

### 3.2. Templating Agents

Heterogeneous porphyrin assemblies have been obtained by the self-assembly of macrocycles with organic or inorganic templates, exploiting the specific interaction among porphyrin macrocycle and the template of interest.

**Scheme 3:**
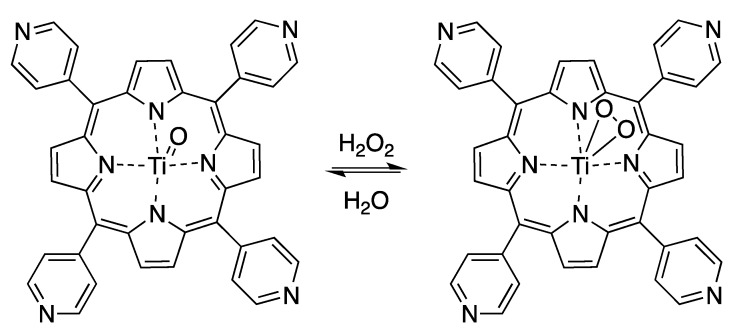
Schematic drawing for the redox process of the TiOTPyP and its monoperoxo complex of TiO_2_TPyP [60].

For example, coordination interactions between the peripheral pyridyl groups of OTiTPyP and PdCl_4_^−2−^ have been exploited to obtain the layer-by-layer deposition of hybrid thin films onto quartz or gold substrates [60]. UV–Vis spectra of these layers showed a 12 nm red shift of the porphyrin Soret band with respect to the solution spectra, while AFM characterization carried out onto gold surface showed the formation of irregular domains, which increased in dimension with the deposition of further layers. The obtained layers were tested for hydrogen peroxide sensing; the hybrid layers deposited onto a quartz substrate showed a 6 nm red shift of the OTiTPyP when immersed into a H_2_O_2_ water solution due to the formation of the peroxo species (Scheme 3). This change is completely reversible just by washing the quartz slide with water or simply exposing it to the air. The layers deposited onto Indium Tin Oxide (ITO) electrodes also generated photocurrents when irradiated with a Xe arc lamp.

**Figure 12:**
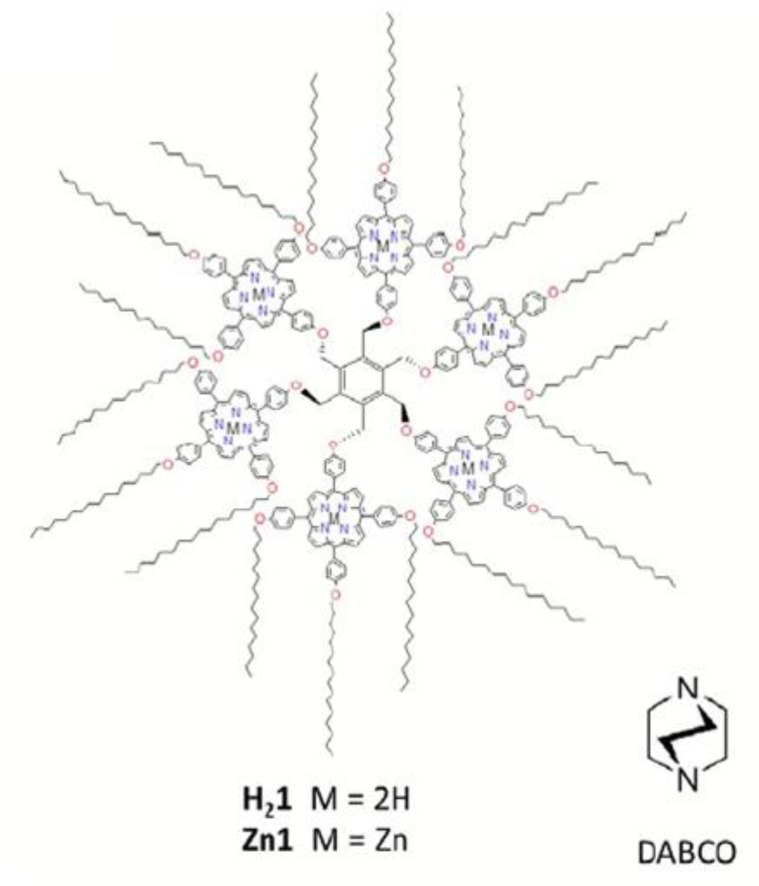
Structure of porphyrin hexamers H21 and Zn1, and of the bidentate ligand DABCO. Adapted with permission from [61]. Copyright 2018, Royal Society of Chemistry.

Taking advantage of the coordination interactions, a diamine, 1,4-diazabicyclo [2,2,2] octane (DABCO), was used to induce the self-assembly of the Zn porphyrin hexamer in CHCl_3_ solution (Figure 12) [61].

While a 1:3 hexamer:DABCO ratio drove the interaction to dimeric species by intermolecular sandwich complexes, the increase of DABCO equivalents led to the formation of polymeric rod like aggregates, as demonstrated by UV–Vis, Nuclear Magnetic Resonance (NMR) spectroscopy, and small angle neutron scattering characterization.

In a different approach, sodium alkyl sulfate surfactants with different alkyl chain lengths (C_14-16-18_OSO_3_Na) have been mixed in a stoichiometric ratio with positively charged meso-tetrakis(4-N-methylpyridinium)porphyrin in water to obtain supramolecular aggregates [62]. The aim of the work was to improve the cellular uptake of the cationic porphyrin, and consequently its biological activity as anticancer agents. The choice of three different surfactants was to investigate the influence of the hydrophobic effect induced by the different alkyl chain lengths on the aggregate formation. When mixed in solution, the porphyrin and alkyl surfactant formed ion pairs and NaCl.

**Figure 13:**
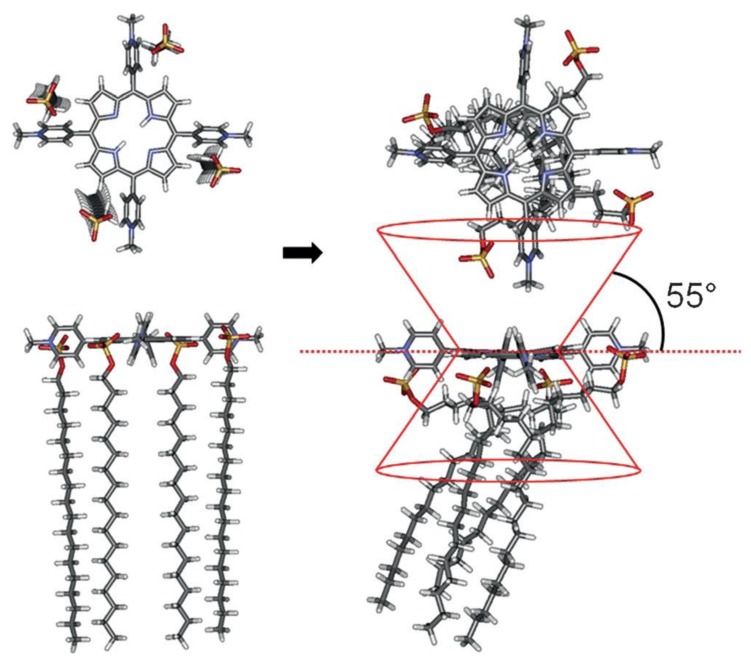
Modeling of the cationic porphyrin–C_18_OSO_3_Na ion pair structure in water reported by Pratviel et al. Reprinted with permission from [62]. Copyright 2015, Wiley.

The UV–Vis absorption spectra of the ion pairs showed a blue-shift of the porphyrin Soret band, which seems to indicate the formation of H-aggregates, although this geometry is connected to the exposure of surfactant alkyl chains to the water solution. Fluorescence spectra showed a splitting of the porphyrin emission, then used to determine the critical aggregation concentration (CAC), which was connected to the length of surfactant alkyl chain. The longer the alkyl chain, the smaller the CAC, leading to more stable aggregates. The TEM images showed spherical supramolecular aggregates, with an average diameter of 160 nm, in accordance with the DLS measurements. NMR characterization needed the addition of a further equivalent of the surfactant as the pristine aggregates are NMR silent; the signal broadening confirmed the formation of aggregates, while DOSY experiments gave a calculated hydrodynamic diameter of 8 nm, much smaller than that indicated by TEM images. NMR studies gave indication on the geometry of the porphyrin-surfactant ion-pair, with the long alkyl chain oriented below the porphyrin plane. This geometry was also confirmed by molecular modeling calculation, reported in Figure 13 for the cationic porphyrin–C_18_OSO_3_Na system in water.

Poly-electrolyte species have also been exploited to interact with ionic porphyrins in water solutions.

The assembly of anionic tetrasulfonatophenylporphyrin onto core−shell polyelectrolyte microcapsules (PECs) of poly(styrenesulfonate) (PSS) and poly(allylamine hydrochloride) (PAH) coating microparticles of CaCO_3_ has been reported [63]. The studies have been carried out at two different pH values, 3.0 and 1.5, to evaluate its influence on the aggregation behavior. By using different spectroscopic techniques and confocal fluorescence microscope images, it was demonstrated that at pH 3, the zwitterion form of TPPS formed needle like aggregates, radially oriented from the PEC surfaces. The CaCO_3_ core was necessary for this porphyrin assembly because hollow polyelectrolyte microcapsules did not show the formation of TPPS J-aggregates. Lowering the pH, the CaCO_3_ core of PECs was unstable and this also destabilized the TPPS aggregate.

**Figure 14:**
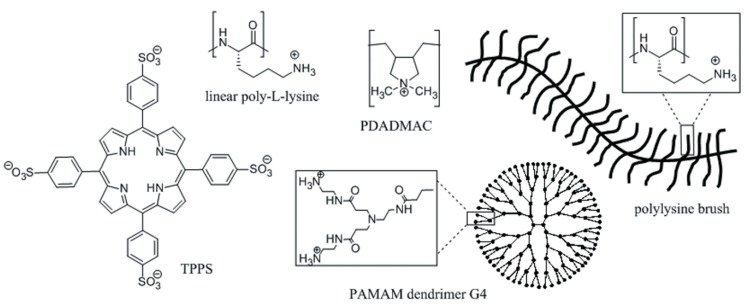
Building blocks used in this study. Positively charged polyelectrolytes of different architecture are combined with TPPS to form nanoscale assemblies in aqueous solution. Reprinted with permission from [64]. Copyright 2017, Wiley.

The influence of the polyelectrolyte structure on TPPS assembly has been studied by Grohn and Fruhbeisser [64]. Different polyelectrolytes have been systematically studied, changing both the morphology and the mass of the polyelectrolyte, going from a dendrimer to a polylysine brush through linear polylysines of different lengths (Figure 14) by using various spectroscopic techniques. This study demonstrated that the polyelectrolyte structure is more important in driving the architecture of the resulting supramolecular aggregates than the relative mass of the polyelectrolyte, although a minimum length should be present. Furthermore, ζ-potential measurements revealed that the surface charge density of the aggregate drives the assembly process. All this information is important for the rational design of these supramolecular assemblies.

TPPS has also been exploited for assembling with a short cationic peptide, with the aim to explore its potential application in light harvesting devices [65]. The assembly was carried out in solution at pH = 2.5, where the zwitterionic form of TPPS is present. The cationic peptide assembles as nanofibers in acidic solution and then TPPS was added to the peptide aggregates, and the cationic nature of the nanostructure can drive the aggregation of TPPS units. The addition of TPPS induced the formation of green precipitates and the TEM images show changes in the diameters of the fibers, which were no more uniform and had significant bundles and entanglement among them. UV–Vis characterization of the assemblies demonstrated the formation of TPPS J-aggregates onto the fiber surfaces. When deposited onto the ITO electrodes, the aggregates showed stable photocurrent signals when irradiated with a Xe lamp. Both the influence of TPPS concentration and solution pH were investigated, demonstrating that the low pH is necessary to induce the assembly of TPPS J-aggregates, while an optimal porphyrin/peptide ratio was needed to obtain the highest photocurrent response.

An intriguing property of porphyrin self-assembly that has been intensively studied is related to the possibility of obtaining aggregates featuring chirality, even with achiral starting macrocycles. These chiral supramolecular aggregates can be obtained by different approaches such as physical effectors like vortex, magnetic field, etc., or by using chiral templating agents [31]. 

One of the most used porphyrin is the anionic, water soluble TPPS and for this reason, chiral acids like tartaric acid have been successfully exploited to obtain chiral assemblies [66]. Quite recently, the formation of TPPS chiral aggregates induced by tartaric acid has been investigated in ethanolic solution [67]. Unlike water, where the formation of TPPS zwitterionic diacid is obtained by the addition of strong acid, in ethanol, tartaric acid was used to form both the TPPS diacid and to drive aggregation by hydrogen-bonding interaction of the not completely dissociated carboxylic groups of the acid and the sulfonate substituents of TPPS.

Following the evolution of the solution upon an increasing amount of l-tartaric acid, the presence of TPPS free base was observed (UV–Vis spectroscopy) at low acid concentration. When l-tartaric acid was in a 10-fold excess, the porphyrin diacid started to form, being in equilibrium with the corresponding free base. In these conditions, no aggregates were formed, while the formation of the ZnTPPS complex was observed, by extraction of Zn ions from the glass container. A further addition of l-tartaric acid showed the rather slow formation of aggregates and the demetallation of the Zn complex. It is interesting to note that in the same conditions, the addition of d-tartaric acid did not induce aggregate formation even after a week. This difference was confirmed when the starting TPPS concentration was increased, in order to favor porphyrin aggregation. Additionally, in this case, a kinetic difference was observed between the two tartaric acid enantiomers, with the l-tartaric acid more prompt to induce TPPS aggregates. This kinetic discrimination resulted in a higher effectiveness of d-tartaric acid to communicate the chiral information to TPPS aggregates, as shown by the CD spectra. The aggregates by l- and d-tartaric acids showed mirrored bands, although their intensities were higher for d-enantiomer; and RLS measurements confirmed the higher size of d-tartaric acid/TPPS aggregates than that of the enantiomeric counterpart.

**Figure 15:**
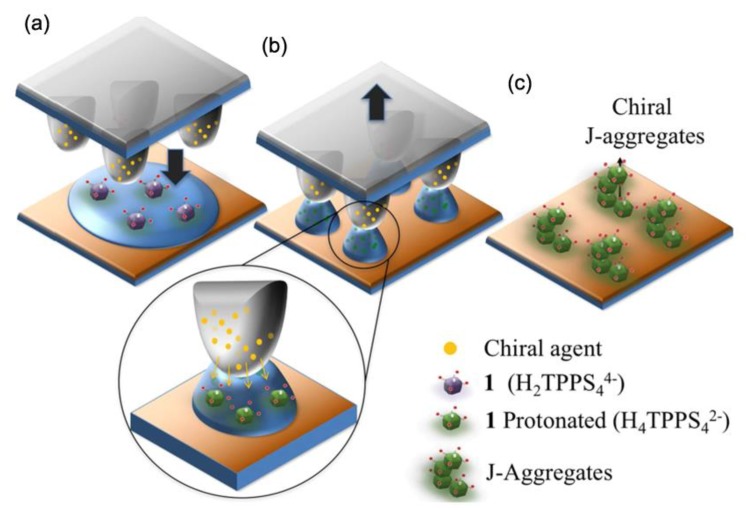
Scheme of the process. (**a**) A stamp pre-loaded with tartaric acid was placed over a film of a water solution spread onto the substrate. (**b**) On solvent evaporation, the solution stayed pinned only to the protrusions, while the region in between the protrusions remained free of solution. During this stage, PDMS stamp releases the chiral agent (tartaric acid) due to osmotic effect. (**c**) When the solution becomes supersaturated, aggregates form in the box shaped between the stamp protrusions. Reprinted from OPEN ACCESS [68].

The tartaric acid templating effect has been exploited to pattern chiral TPPS aggregates onto solid surfaces by soft lithographic method [68]. The process adopted is reported in Figure 15.

A PolyDiMethylSiloxane (PDMS) stamp was immersed in an ethanol solution of tartaric acid; the PDMS swelled, absorbing the solution and the tartaric acid templating agent. This stamp was placed in contact with an aqueous solution of TPPS, spread on a silicon or glass substrate. The tartaric acid diffused in the solution and the TPPS zwitterionic diacid started to form. Afterward, the water evaporated, leaving the solution in contact only in the stamp protrusions. When the solution reached supersaturation conditions, the porphyrins started to aggregate in this region. The process allowed the chirality to transfer to the TPPS aggregates and their patterning onto the solid surface. This approach demonstrates that a soft lithographic approach is possible and allows the deposition of supramolecular chiral aggregates patterning a solid surface.

The influence of the coordination of the Zn ion extracted by TPPS from the glass container, on the porphyrin aggregation pathway has been investigated in detail [69]. Thermal treatment of the TPPS solution was performed to have the monomeric porphyrin, but this process also enhanced the formation of ZnTPPS, as indicated by both UV–Vis and fluorescence characterization. ZnTPPS underwent demetallation in the acidic condition necessary for porphyrin aggregation, but strongly influenced the kinetics of the assembly, reducing the kinetic constant of the process. This variation was however accompanied by defined CD spectra for the solution containing ZnTPPS. This feature was confirmed by treating the TPPS in a plastic container, so not containing Zn ions, and then this solution was used as a blank reference for subsequent aggregation. A similar solution was added to the Zn ions and then subjected to aggregation; it was observed that the aggregate from the blank solution showed a negative bisignated Cotton band, while aggregates from the Zn ion added solution showed a more intense, positive Cotton band. Once more, the reduced aggregation rate allowed a more effective transmission of the chiral information in the developing TPPS assemblies.

The aggregation of ZnTPPS in acidic conditions in the presence of polyelectrolytes has also been investigated [70]. In the case of the ZnTPPS solution, when pH = 1.5 is reached, an immediate demetallation leading to the zwitterionic TPPS diacid occurs, but no aggregation is observed within the first 6 h, confirming the inhibition activity of the Zn ion in the TPPS aggregation. When the effect of reducing pH was carried out in the presence of poly-d-glutamate, an anionic polyelectrolyte, no differences were observed. A different behavior was observed when the same experiments were carried out in the presence of poly-Lysine (PLL), a cationic polyelectrolyte. At pH = 1.5, a clear delay of ZnTPPS demetallation was observed, however, UV–Vis spectra after one-hour of this solution showed the formation of TPPS J-aggregates. These results are both attributed to the PLL action, which delayed the ZnTPPS demetallation because it can compete in proton binding, but can also act as a template for negatively charges TPPS diacid, therefore driving its aggregation.

**Figure 16:**
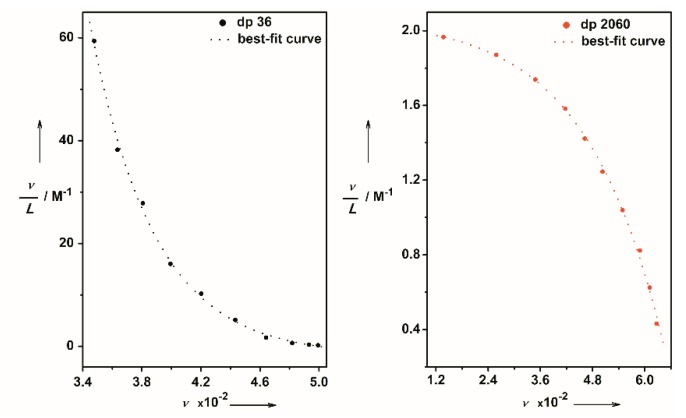
Binding isotherm for the titration of ZnTPPS with poly-l-lysine dp36 (**left**) and dp 2060 (**right**); [ZnTPPS] = 2 μM, 7.5 μM < [PLL] < 30 μM, 25 °C, pH 7. Adapted with permission from [71]. Copyright 2018, Wiley.

The influence of PLLs of various lengths has been studied in detail [71]. When ZnTPPS and PLLs with different polymerization degrees (dp), ranging from dp 36 to 2060, were mixed in solutions of neutral pH, the shortest PLL (dp 36) chain induced the formation of ZnTPPS dimers, while longer PLL chains (dp 2060) drove the formation of hybrid ZnTPPS-PLL aggregates, as evidenced by optical characterization. Titration of ZnTPPS with the two PLLs was carried out, showing different Scatchard plots that evidenced non-cooperative binding with the short chain PLL, and cooperative binding for the long chain PLL (Figure 16).

This result gives indication of a different binding pathway for the two PLLs. In the short PLL chain, the presence of few aggregation sites resulted in the formation of porphyrin dimers, and the formation of the aggregate hinders the formation of other dimers because of mutual steric hindrance. For the long chain, the binding of a TPPS unit increases the affinity of the others by electrostatic and solvophobic effects.

**Figure 17:**
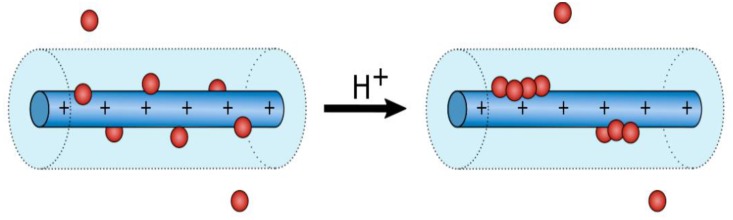
Schematic drawing of a pH-induced self-aggregation process onto a polyelectrolyte mold. Adapted with permission from [71]. Copyright 2018, Wiley.

When HCl was added to reduce the solution pH to 1.5 value, the formation of the TPPS diacid was immediately observed in the UV–Vis spectrum upon acid-catalyzed demetallation. Afterward, the TPPS diacid started to aggregate, as evidenced for the appearance in the UV–Vis spectrum of the characteristic band of J-aggregates, but the kinetics were strongly dependent on the PLL chain length. In the case of short chain PLL, the aggregation step was fast, and the system quickly reached a stationary state, not showing further evolution with time. Conversely, the long chain PLL showed a slower evolution, but the intensity of the TPPS aggregate band was significantly more intense than that of the short chain PLL. CD spectra also confirmed this behavior because the spectrum observed in the case of the short chain PLL was time-independent, while in the case of the long chain PLL, it evolved during time, reaching much more intense signals at the end. The RLS spectra also indicated a higher size for the aggregates obtained with long chain PLL. All these data suggest that the slow growth of the TPPS aggregates obtained in the presence of long chain PLL allowed a more efficient transmission of the template chiral information (Figure 17).

**Scheme 4:**
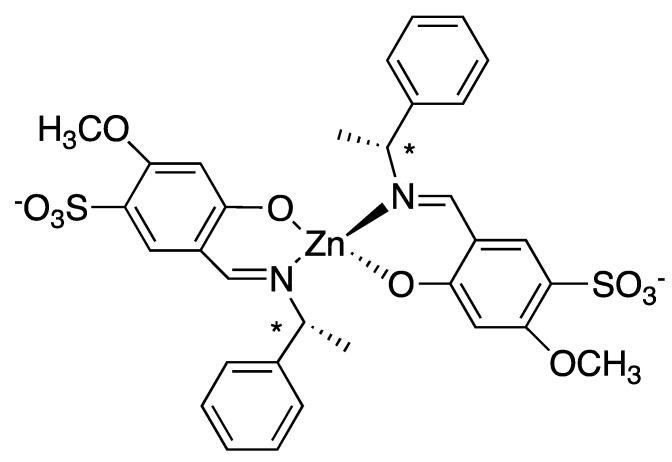
Molecular structure of the chiral Zn(II)Schiff-base complexes [72].

Chiral Zn(II)Schiff-base complexes (Scheme 4) have been exploited as template to induce the chiral assembly of cationic 5,10,15,20-tetrakis (*N*-methylpyridynium-4-yl)porphyrin (TNMePyP) and anionic CuTPPS [72].

**Figure 18:**
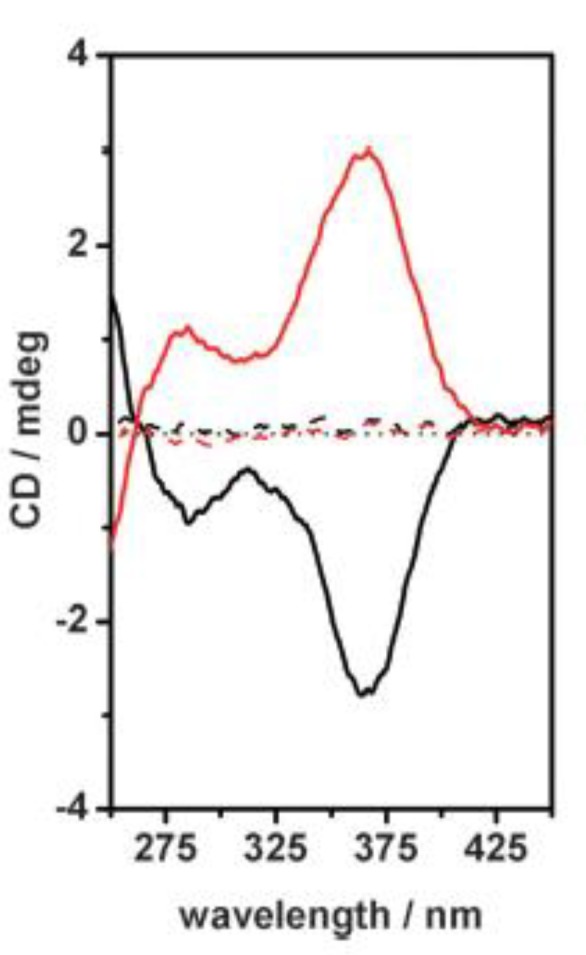
CD spectrum of chiral heteroaggregates obtained by D’Urso et al. Adapted with permission from [72]. Copyright 2016, Royal Society of Chemistry.

The hierarchical character of the assembly is demonstrated by the stepwise addition of TNMePyP to a solution containing the Zn Schiff base, and the final addition of CuTPPS allows the formation of heteroaggregate featuring chirality, as evidenced by a bisignated CD spectrum (Figure 18). No CD signal was obtained when the Zn Schiff-base complex was added to a solution containing the preformed porphyrin heteroaggregate.

**Figure 19:**
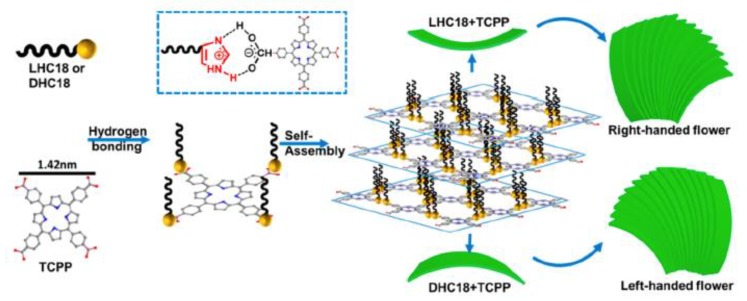
Schematic illustration of the interaction modes between histidine derivatives and TCPP. Reprinted with permission from Ref [73]. Copyright (2017) American Chemical Society.

Interestingly, the heteroaggregates showed chiral memory effects, as indicated by the persistency of the ICD effect, even after removal of the Zn-complex template, which could be simply accomplished by the incubation of the solution in water for 24 h due to the instability of this species. While the Schiff base chiral ligands also had the ability to drive the chiral aggregation of TNMePyP and CuTPPS, they were much less effective than the corresponding Zn complexes because they formed less ordered aggregates, as evidenced by less intense CD spectra.

l- and d-histidine has been exploited as templates to obtain chiral structures by assembling with 5,10,15,20-tetrakis(4-carboxyphenyl)porphyrin (TCPP) in DMF/H_2_O solution [73]. TCPP and l-histidine were dissolved by warming in DMF and then water was added to induce aggregation. The porphyrin/amino acid ratio determined the morphology of the resulting aggregate; it was necessary to have a TCPP/histidine 1:4 ratio to obtain chiral nanoflowers, and the handedness was determined by the histidine enantiomer (Figure 19).

The histidine enantiomer was able to transfer the chiral information to the hybrid assembly; to confirm this result, the aggregation was carried with a racemic mixture of the amino acids, in this case, nanorod and flower morphologies were obtained, but without a clear chiral orientation. CD spectra of the aggregates showed mirror spectra for the aggregates obtained with the enantiomeric pair, again supporting the role of histidine in transferring the chiral information to the aggregate.

The chiral nanoflowers showed a superhydrophobic character, which was used to investigate the potential chiral recognition toward different amino acids. It was found that this chiral nanoflower showed a different variation of the contact angle in the case of solutions of aspartic acid enantiomers, demonstrating promising enantioselective properties.

**Scheme 5:**
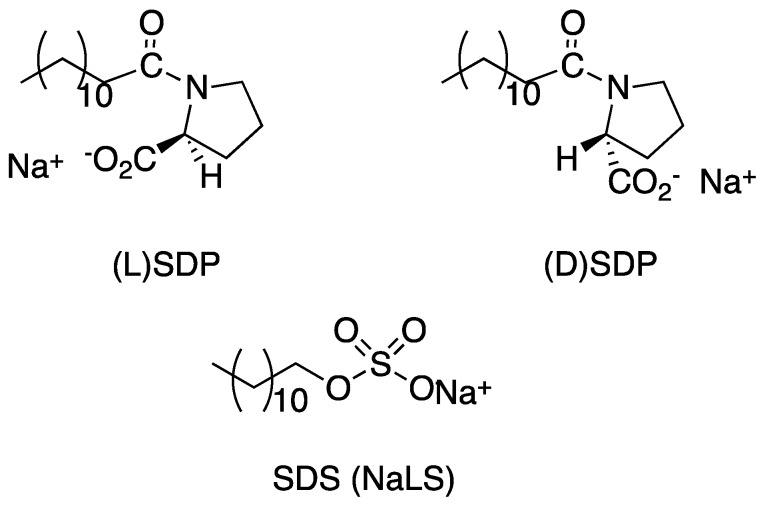
Molecular structures of the surfactants used in aggregation studies of porphyrin **10c** [74].

The influence of chiral surfactants on the aggregation of the cationic porphyrin bearing a chiral proline substituent **10c** (Figure 8a) has been investigated [74]. l- and d-sodium dodecyl prolinate (SDP) (Scheme 5) in water were added to an absolute ethanol solution of porphyrin, and then water was added to reach the ethanol/water ratio of 1:3 *v*/*v*.

Two different concentrations of the surfactants were investigated. The assembly process was monitored by UV–Vis and CD spectroscopy. At low L-SDP concentration, the solution mixing induced an abrupt drop in the porphyrin Soret band, followed by a slower evolution toward the equilibrium conditions. CD spectroscopy indicated the quick formation of non-chiral aggregates during the first step, which then evolved in supramolecular chiral assemblies by a cooperative process. At higher surfactant concentrations, a different pathway was observed: again, the rapid formation of achiral aggregates was followed by the slow formation of J-aggregates, which differed from those observed at low SDP concentration. The CD spectrum, in fact, showed an inversion of the dichroic bands, with a remarkable increase in the relative intensities. This result seems to indicate a more efficient transfer of the chiral information from SDP to the porphyrin aggregates. This SDP concentration effect has been attributed to a pre-micellar structure present in the more concentrated solution of the surfactant, which are responsible for the more efficient transmission of the stereogenic information. A similar pathway was observed in the case of the D–SPD surfactant, but no inversion of the CD bands was registered; this result indicates the diastereoselective nature of the surfactant–porphyrin interactions, which influence the dissymmetry of the supramolecular structure, but preserve the short-range J-aggregation structure. To further investigate the influence of the surfactant on the chiral aggregation character, the non-chiral SDS surfactant, similar in structure to SDP, was exploited in the same aggregation conditions. In this case, the CD silent spectra demonstrated that the aggregates did not show chirality features. While the chiral SDP surfactants induced a chiral amplification, the non-chiral SDS probably perturbed the proline chiral information, leading to non-specific aggregates.

## 4. Physical Effectors

**Scheme 6:**
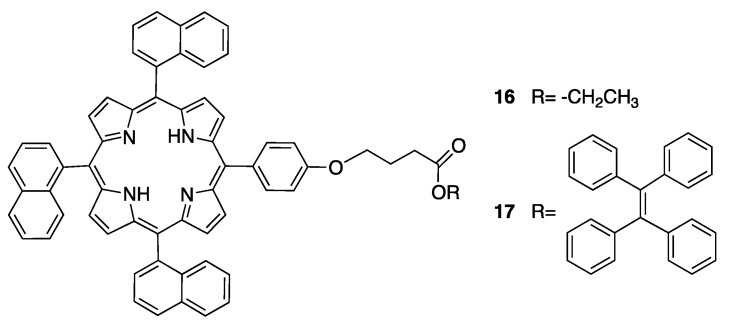
Molecular structures of the porphyrin derivatives studied by Cai et al. [75,76].

A viable alternative to prompt the formation of a nano/micro ordered structure utilizes physical parameter tuning. Recently, Cai et al. showed that a large number of different nanostructures could be obtained by droplet template by regulating the temperature during the self-assembly of porphyrins [75,76]. The first evidence of the efficiency of this *modus operandi* involved the use of porphyrin derivatives of 5-(4-(ethylcarboxypropoxy)phenyl)-10,15,20-tri(naphthyl), and more recently, 4-[4-[10,15,20-tris(naphthyl)-21*H*,23*H*-porphin-5-yl] phenoxy]-4-(1,2,2-triphenylvinyl) phenylbutyrate (Scheme 6, compounds **16** and **17**, respectively). This latter porphyrin has longer conjugated bonds that in turn increases the strength of assembly, allowing for higher fabrication reproducibility.

**Figure 20:**
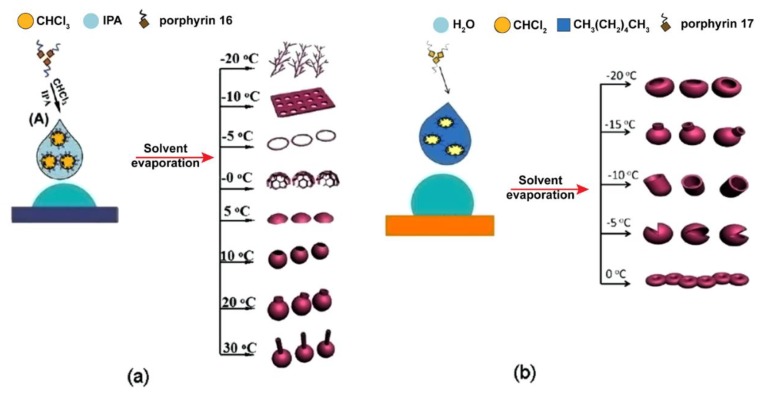
Schematic representation of the temperature-controlled formation of various nanostructures from porphyrin derivatives **16** (**a**) and **17** (**b**) by varying the assembly temperature. Adapted with permission from [75,76]. Copyright 2015 and 2018, Royal Society of Chemistry.

Briefly, the droplet template process begins with the formation of porphyrin vesicles in solution: porphyrin **16** (or **17**) was dissolved in chloroform (dichloromethane) with a concentration of 10^−^^5^~10^−^^6^ mol/L and then the solution was poured into a solvent where porphyrin is poorly soluble such as isopropyl alcohol *(hexane).* As a result, vesicles were formed that possessed an inner hydrophobic core and an outer hydrophilic shell. Subsequently, this suspension was cast onto a water droplet template distributed over a hydrophobic substrate and was left to evaporate at different temperatures. Once the solvent was removed, nanostructured assemblies were obtained in accordance with the assembly temperatures; the resulting morphologies included jar-, flask-, cup-, open smile-/tulip-, and chain-like structures (Figure 20a,b). The temperature affects the stick-slip mode of the three phase contact line of the droplet, producing different nanostructured assemblies. In other words, the balance between the pinning force (resistance force from the substrate) and depinning force (capillary force) determines the shape of the drop during the evaporation process, and as a consequence, the nanoaggregate morphologies. In the case of porphyrin **17**, a large scale fabrication reproducibility was found thanks to the improved assembly strength. Finally, the photoluminescence absolute quantum yield (@375 nm laser excitation) of cup-like nanostructures resulted in being comparable with the one of compound **17** in solution, leading to the possibility of extended applications in optical based devices.

An alternative method to affect the hierarchical structure of porphyrin aggregates utilizes ultrasonic waves in order to increase both the temperature and pressure of the media.

**Scheme 7:**
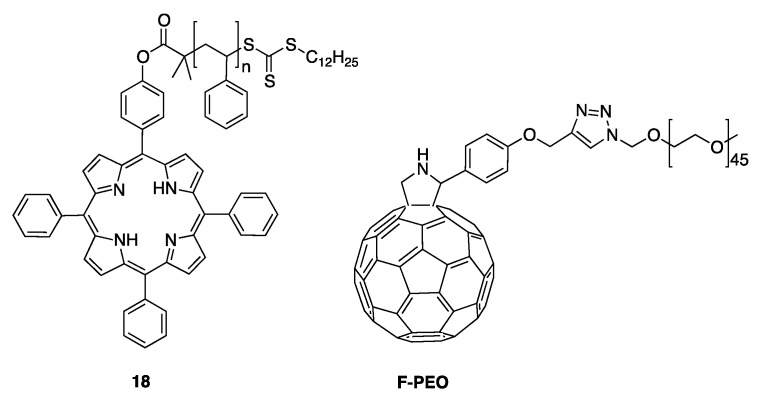
Molecular structures of the porphyrin and fullerene subunits used by Riaz et al. [77].

Recently, Riaz et al. demonstrated a procedure to induce the self-aggregation of porphyrin-fullerene derivatives into polymeric rings when assisted by sonication [77]. In the absence of ultrasonic treatment, monochelic porphyrinic polystyrene **18** and Fullerene-end-capped polyethylene oxide (F-PEO) (Scheme 7) self-assembled into fibrils or nanospheres when mixed in solution without evidencing the formation of nanoring aggregates. Typically, the sonicated assisted procedure consists of mixing porphyrin 18 and F-PEO (1:1 molar ratio) in DMF and stirring for 4 h; the mixed solution is then sonicated for 40 min at room temperature and stirred again for 4 h. Finally, before being characterized, the solution was dialyzed in THF. The TEM and AFM micrographies revealed that nanorings are polydisperse and their dimensions range from 100 to 1500 nm. It is important to emphasize that the sonication period should be carefully optimized since exceeding the energy provided during the aggregation time leads to the formation of vesicles that result in nanospheres. The main advantage of nanoring morphology is the improvement in the optical properties of these structures when compared to the others, since they showed a reduced quenching of fluorescence with respect to the one observed in the case of spheres, probably because interaction between **18** and F-PEO was confined in the 2-D plane of the rings. Although the influence of visible light has been deeply explored in the case of porphyrins since this class of macrocycles are well known photosensitizers and photocatalysts, the influence of visible radiation on the aggregation has not been so explored. The light force can actually drive or prompt the aggregation mechanism in solution, even if the effectiveness of this method is limited since the force exerted by optical trap potential on 1 nm-molecules (such as porphyrin) is orders of magnitude lower than that of thermal agitation energy.

Shirakawa et al. demonstrated that the light induced force may have an effect on the aggregation and disaggregation of TPPS through the absorption changes of the solution [78]. Experiments were performed using different solutions where free base monomers (H_2_TPPS^4−^), diacid monomers (H_4_TPPS^2−^), and J-aggregates of TPPS were predominant, respectively. The diacid monomers solution was prepared by dissolving 16 μmol/L TPPS in deionized (D.I.) water; J-aggregation was obtained by adding 0.4 mL of 1 mol/L hydrochloric acid to the former TPPS aqueous solution, whereas free base monomers were formed by adding 0.2 mL of an aqueous solution of sodium hydroxide at a concentration of 0.1 mol/L. It is worth noting that the porphyrin concentrations utilized in the work were lower than the concentration required for the growth of stable aggregates, and the excitation wavelength of the laser beam was 532 nm, in the middle between the B and Q bands of TPPS. Results showed that in the case of monomers, light excitation produced a decrease in the monomer band coupled with an increase of absorption in the red-shift wavelength; in the case of diprotonated-monomers, there was a formation of small J-aggregates (characterized by a peak in the 490 nm region). The variations in absorption spectra were higher than expected (in the 10^−^^3^ range), suggesting the presence of local cofactors like an increase of temperature and changing of liquid viscosity that assists the aggregation process. In the case of the J-aggregates, the visible irradiation produced a blue shift in the 490 nm peak. Indeed, blue-shift induced by electromagnetic field had been rarely observed and in in this case, hypsocromic variation is likely to be due to a rearrangement or dissociation of J-aggregates.

**Figure 21:**
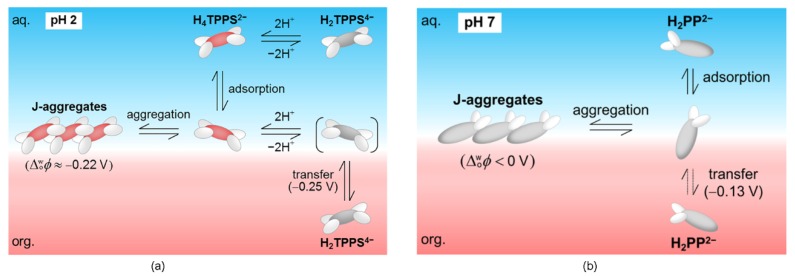
Schematic representation of the aggregation and transfer of (**a**) H_2_TPPS^4−^ and (**b**) H_2_PP^2−^ at the polarized water/CH_2_Cl_2_ interface. Adapted with permission from [79]. Copyright 2017, American Chemical Society.

Yamamoto et al. investigated the possibility of driving the aggregation of TPPS and protoporphyrin IX at the liquid–liquid interface by applying an external potential [79]. The polarization-modulation total internal reflection fluorescence (PM-TIRF) technique was used to study the self-aggregation of former porphyrin at the polarized water/dichloroethane interface (Figure 21). In the case of diacid TPPS, PM-TIRF showed that diprotonated monomers were found in water, whereas free base TPPS was predominant in the organic solution, respectively, and that polarization induced the self-aggregation (J-aggregates) only at the interface of the two media. In the case of H_2_PP^2−^, J-aggregation took a considerably long time (ca. 20 min) before reaching equilibrium. As in the case of H_2_PPS^2−^, aggregation was induced by external potential only at the organic/water interface and remarkably, J-aggregate features appeared even at dilute concentrations (10^−^^5^ M). However, increasing the concentration monomer began to become dominant over the aggregation, most likely due to the upstanding orientation of the former molecules, which limits the area available for the aggregates. The H_2_PP^2−^ system was also studied in the presence of phospholipid-adsorbed water/CH_2_Cl_2_ and showed that the potential induced aggregation state of the protoporphyrin was effectively inhibited by the glycerophospholipids. This latter experiment was aimed to study the aggregation process by mimicking a biological environment such as tissues.

Ribó’s group have reported many papers illuminating the effect of hydrodynamic forces of a flow on J-aggregation of amphiphilic porphyrin derivatives [80]. Thanks to these studies, it is nowadays well-established that the size and shape of J-aggregates is influenced by hydrodynamic forces since they can act on both the mechanisms and growth rates and mechanically shape the large enough J-aggregate particles. The same group recently showed that hydrodynamic forces of stirring not only affected the self-assembly processes of 4-sulfonatophenyl *meso*-substituted porphyrins, but also modified the size and shapes of the formed nanoparticles by elastic and plastic behavior. Cryo-TEM imaging was decisive in resolving the contradictory descriptions given in previous works of the formed particles as ribbons, nanotubes, or nanorods: indeed, the authors found that in solution, the structures are hollow nanotubes that become ribbons through collapse when deposited on dry substrates for PFM analysis. These soft-matter nanoparticles either showed elastic or plastic behavior. In detail, plasticity features in the ribbons obtained upon nanotube collapse and in viscous concentrated nanotube solutions, whereas elastic behavior is characterized by the dilute nanotube solutions. When these solutions were subjected to sonication or to strong shear hydrodynamic forces, a strong Tyndall effect was visible together with the typical absorptions of J-aggregates, indicating scattering in the visible region indicative of the disruption of the monolayered nanotubes into small particles. In the PFM images, these small particles appeared as domains of particles welded to one another, presumably generated by the collapse of large colloidal particles. Interestingly, the UV–Vis spectra were almost the same before and after sonication, even the bands located at 420 and 491 nm showed a lower extinction coefficient after treatment. This was explained as a lower RLS contribution in the case of small particles with respect to nanotubes. At 10 months after sonication, the formation of long, regular nanotubes (seen as ribbons by PFM analysis) with a concomitant increase of the RLS contribution to the excitonic band at 491 nm was detected. This finding demonstrated the ripening or repairing effect of the nanoparticle morphology.

## 5. Conclusions

“Who knows where the time goes”, beautifully sang Sandy Denny in one of the legendary masterpieces of the UK folk era (Fairport Convention’s Unhalbricking; Island Records 1969). This title would be, in a more pragmatic yet important way, and without disturbing the astrophysical definition, the *leitmotiv* of this review, which focused on self-assembled structures of porphyrin derivatives. Without any doubt, the results discussed in this review show the countless opportunities of obtaining complex porphyrin-based structures featuring captivating properties for exploitation in the achievement of functional materials for use, for example, as light-harvesting or photocurrent generation systems in molecular recognition or sensing fields. The fine tuning of the properties of the described aggregates is made possible by a florilegium of either internal or external stimuli such as solvent polarity, pH, ionic strength, and templates. However, despite the consolidated methodologies developed to form different porphyrin-based nanostructures in both solutions and in the solid state, concrete applications still lag behind. Surely, in the last few years we have observed the ever-growing supporting role of several microscopic techniques (AFM, SEM, TEM) to practically visualize the prepared structures. The use of these techniques has helped both the systematic exploration of experimental conditions to realize morphologically controlled assemblies of reproducible size and the understanding of the structure–function relationship wherever they were tested in specific applications. Of course, multiple limitations concerning, among others, the robustness and versatility of the aggregates still have to be overcome for their efficient real-life implementation in a device. The challenge will especially lie in this last aspect in the near future.
